# Glutamine-mediated epigenetic regulation of cFLIP underlies resistance to TRAIL in pancreatic cancer

**DOI:** 10.1038/s12276-024-01231-0

**Published:** 2024-04-30

**Authors:** Ji Hye Kim, Jinyoung Lee, Se Seul Im, Boyun Kim, Eun-Young Kim, Hyo-Jin Min, Jinbeom Heo, Eun-Ju Chang, Kyung-Chul Choi, Dong-Myung Shin, Jaekyoung Son

**Affiliations:** 1 Department of Biochemistry and Molecular Biology, Brain Korea 21 Project, Asan Medical Center, University of Ulsan College of Medicine, Seoul, 05505 South Korea; 2 Department of Cell and Genetic Engineering, Brain Korea 21 Project, Asan Medical Center, University of Ulsan College of Medicine, Seoul, 05505 South Korea

**Keywords:** Cancer metabolism, Apoptosis

## Abstract

Tumor necrosis factor-related apoptosis-inducing ligand (TRAIL) is a promising anticancer agent because it kills cancer cells while sparing normal cells. However, many cancers, including pancreatic ductal adenocarcinoma (PDAC), exhibit intrinsic or acquired resistance to TRAIL, and the molecular mechanisms underlying TRAIL resistance in cancers, particularly in PDAC, remain unclear. In this study, we demonstrated that glutamine (Gln) endows PDAC cells with resistance to TRAIL through KDM4C-mediated epigenetic regulation of cFLIP. Inhibition of glutaminolysis significantly reduced the cFLIP level, leading to TRAIL-mediated formation of death-inducing signaling complexes. Overexpression of cFLIP dramatically rescued PDAC cells from TRAIL/Gln deprivation-induced apoptosis. Alpha-Ketoglutarate (aKG) supplementation significantly reversed the decrease in the cFLIP level induced by glutaminolysis inhibition and rescued PDAC cells from TRAIL/Gln deprivation-induced apoptosis. Knockdown of glutamic-oxaloacetic transaminase 2, which facilitates the conversion of oxaloacetate and glutamate into aspartate and aKG, decreased aKG production and the cFLIP level and activated TRAIL-induced apoptosis. AKG-mediated epigenetic regulation was necessary for maintaining a high level of cFLIP. Glutaminolysis inhibition increased the abundance of H3K9me3 in the cFLIP promoter, indicating that Gln-derived aKG production is important for Jumonji-domain histone demethylase (JHDM)-mediated cFLIP regulation. The JHDM KDM4C regulated cFLIP expression by binding to its promoter, and KDM4C knockdown sensitized PDAC cells to TRAIL-induced apoptosis. The present findings suggest that Gln-derived aKG production is required for KDM4C-mediated epigenetic regulation of cFLIP, which leads to resistance to TRAIL.

## Glutamine’s Role in TRAIL Resistance: Epigenetic Regulation Unveiled

Pancreatic ductal adenocarcinoma (PDAC), a severe form of pancreatic cancer with a low survival rate, was the focus of this study. The aim was to understand why PDAC is resistant to TRAIL-induced programmed cell death (apoptosis). The study found that PDAC cells need glutamine (a type of amino acid) to be resistant to TRAIL-induced apoptosis. Without glutamine, these cells are more likely to undergo TRAIL-induced apoptosis due to a decrease in a protein called cFLIP. This protein’s regulation involves a process with glutamine-derived alpha-ketoglutarate (aKG), a chemical compound. The study also discovered that an enzyme called KDM4C helps regulate cFLIP. The results suggest that focusing on glutamine metabolism could be a potential TRAIL-based therapy for PDAC.

## Introduction

Pancreatic ductal adenocarcinoma (PDAC) is the most prevalent neoplastic tumor of the pancreas; it accounts for 90% of all pancreatic cancers and is an extremely aggressive tumor with an expected 5 year survival rate of 8%^1^. Patients with PDAC have a dismal prognosis because the disease is often diagnosed at a late stage, and surgical resection is no longer possible. In addition, PDAC often exhibits resistance to chemotherapy and radiotherapy^2^, resulting in one of the lowest survival rates among all malignancies; moreover, the 5 year survival rate of PDAC patients has improved little in recent decades. The poor survival rate of patients with PDAC and the lack of effective treatments underscore the need to improve our understanding of the molecular mechanisms underlying tumor resistance to apoptotic cell death to identify effective therapies for PDAC.

Apoptosis is a regulated programmed cell death process that is distinct from other forms of cell death, such as autophagy and necrosis. It eliminates cells that are no longer needed or are damaged, thereby maintaining homeostasis in multicellular organisms. The regulation of cell death is critical, and inappropriate apoptosis contributes to the development of many diseases, such as neurodegenerative diseases, autoimmune disorders, and many types of cancer^3,4^. Certain oncogenic mutations result in inactivation of apoptosis during tumor initiation and progression, and many studies have aimed to develop anticancer agents that promote the effective elimination of cancer cells via apoptosis. Although many anticancer agents have shown potential in the early stages of drug development, they have failed in clinical trials because of their very unfavorable toxicity profiles^5^. TNF-related apoptosis-inducing ligand (TRAIL; also known as Apo2L) is a proapoptotic ligand that belongs to the TNF family^6^. TRAIL has emerged as a promising anticancer therapeutic agent because it can specifically induce apoptosis in cancer cells with little to no toxicity to normal cells^7^. Despite its advantages as an anticancer therapeutic agent, intrinsic or acquired resistance to TRAIL limits the success of TRAIL-based therapies. Thus, extensive research efforts are needed to elucidate the mechanisms underlying TRAIL resistance, as this knowledge may facilitate the development of new and effective TRAIL-based strategies.

Metabolic reprogramming in cancer has become a topic of interest in the past decade. The reprogramming of cancer cell metabolism is important for meeting the increased biosynthetic needs associated with abnormal cell proliferation^8^. The Warburg effect is the classical example of metabolic reprogramming in cancer cells. In particular, pancreatic cancer cells are highly dependent on glutamine (Gln) metabolism because Gln is an important nitrogen donor for the synthesis of hexosamine, nonessential amino acids (NEAAs), fatty acids, and antioxidants. Additionally, under extreme reactive oxygen species (ROS) accumulation, cancer cells rewire the Gln metabolic pathway to maintain redox homeostasis via NADPH production through glutaminolysis^9^. Gln is also converted to alpha-ketoglutarate (aKG) by multiple enzymes to enable ATP production through the tricarboxylic acid (TCA) cycle^10,11^. Epigenetics, which involves the chemical modification of DNA or histones, is a mechanism linking cellular metabolism and gene expression. Because metabolites act as cofactors required by epigenetic enzymes, metabolic reprogramming in cancer cells affects the regulation of gene expression^12^. Conversely, dysfunction of epigenetic processes can modify metabolism by directly affecting the expression of metabolic enzymes, which may lead to alterations in the metabolome^13^. The modification of DNA and histones is regulated by the activity of DNA demethylases and histone demethylases, respectively. DNA demethylation is catalyzed by ten-eleven translocation methylcytosine dioxygenase (TET)^14^, whereas the lysine-specific protein demethylase (KDM1) family and Jumonji C-domain-containing (JMJD) family enzymes catalyze histone demethylation. TETs and JMJDs both require aKG as a cofactor^15^. Most of the aKG in PDAC cells is derived from Gln, as evidenced by ^13^C labeling^11^. Therefore, crosstalk between epigenetic modifications and Gln metabolism plays a critical role in PDAC tumorigenesis.

In this study, for the first time, we demonstrate the Gln-mediated epigenetic regulation of cFLIP, which endows PDAC cells with resistance to TRAIL. Gln deprivation significantly decreased the cFLIP level, and knockdown of cFLIP, which is overexpressed in pancreatic cancer, increased susceptibility to TRAIL. Failure to sustain glutamic-oxaloacetic transaminase 2 (GOT2)-mediated Gln-derived aKG production led to a dramatic reduction in cFLIP expression. The suppression of Gln-derived aKG production inhibited KDM4C-mediated epigenetic regulation of cFLIP expression, leading to TRAIL-induced apoptosis. The present findings suggest that targeting Gln metabolism is a promising TRAIL-based therapeutic strategy.

## Materials and methods

### Cell culture

Cell lines were acquired from the American Type Culture Collection or the German Collection of Microorganisms and Cell Cultures and tested regularly for mycoplasma contamination. The cells were maintained in a humidified atmosphere containing 5% CO_2_ at 37 °C in Dulbecco’s modified Eagle’s medium (DMEM; Thermo Scientific, Waltham, MA, USA) supplemented with 10% fetal bovine serum (FBS), 100 U/mL penicillin, and 100 μg/mL streptomycin (Thermo Scientific). For glucose or glutamine deprivation, glucose (G7021), glutamine (G8540), and DMEM without glucose and glutamine (D5030) were obtained from Sigma‒Aldrich (St. Louis, MO, USA).

### Reagents and antibodies

Dimethyl 2-oxoglutarate (hereafter referred to as αKG; 349631), BPTES (SML0601), 2-deoxy-D-glucose (2DG; D8375), N-acetyl-L-cysteine (NAC; A9165), reduced L-glutathione (GSH; G6013), epigallocatechin gallate (EGCG; E4143), and aminooxyacetic acid (AOA; C13408) were obtained from Sigma‒Aldrich. z-VAD-fmk (fmk001) was purchased from R&D Systems (Minneapolis, MN, USA). 5-Aza-2’-deoxycytidine (5-aza; 2624) and JIB04 (4972) were obtained from Tocris Bioscience (Bristol, United Kingdom). TRAIL (TRA0801) was purchased from NKMAX (Seongnam, Republic of Korea). Tetramethylrhodamine ethyl ester (TMRE) (T669) was obtained from Thermo Fisher Scientific. Antibodies against BCL2 (2870), BCL-xL (2764), BAD (9292), BAK (6947), BAX (2772), BID (2002), Puma (4976), BIM (2933), caspase-3 (9662), caspase-8 (9746), caspase-9 (9502), PARP (9542), MCL-l (5453), XIAP (2042), and survivin (2808) were purchased from Cell Signaling Technology (Beverly, MA, USA). The anti-FLIP antibody (ALX-804-961-0100) was acquired from Enzo Life Science. Antibodies against β-Actin (sc-47778) and CIAP1 (sc-7943) were purchased from Santa Cruz Biotechnology (Dallas, Texas, USA). Anti-TET1 (GTX124207) and anti-TET3 (GTX121453) antibodies were obtained from GeneTex (Irvine, CA, USA). The anti-TET2 antibody (ab94580) was purchased from Abcam (Cambridge, UK). Antibodies against DR4 (1139) and DR5 (2019) were obtained from ProSci, Inc. (Poway, CA, USA). The anti-FADD antibody (610399) was obtained from BD Pharmingen (Franklin Lake, NJ, USA). The antibody against KDM3A (12835-1-AP) was purchased from Proteintech (Rosemont, IL, USA). Antibodies against KDM4A (A300-861A), KDM4C (A300-885A), and KDM5B (A301-813A) were obtained from Thermo Fisher Scientific.

### Annexin V/propidium iodide (PI) assay

Apoptotic cell death was evaluated by an Annexin-V/fluorescein isothiocyanate (FITC)/PI assay. Cells were harvested by trypsinization, washed with PBS, and pelleted cells were resuspended in Annexin V binding buffer (10 mM HEPES, pH 7.4, 140 mM NaCl, and 2.5 mM CaCl_2_) and labeled with FITC and PI. FITC- and/or PI-labeled cell populations were quantified and analyzed on a flow cytometer (Beckman-Coulter).

### Oxygen consumption rate measurement

Oxygen consumption rate (OCR) was measured using an XF24 extracellular flux analyzer (Seahorse Bioscience, North Billerica, MA, USA). Briefly, cells were plated in a 24-well Seahorse plate and cultured at 37 °C with 5% CO_2_. The medium was replaced the following day with unbuffered DMEM, and the cells were incubated at 37 °C without CO_2_ for 1 h. For OCR measurement, oligomycin, FCCP, and rotenone were added to final concentrations of 0.5 μg/mL, 1 μM, and 1 μM, respectively.

### Quantification of intracellular ATP

The intracellular ATP concentration was measured using an ATP Colorimetric/Fluorometric Assay Kit (BioVision Incorporated, Milpitas, CA, USA) according to the manufacturer’s instructions. Briefly, cells were lysed in 100 μL of ATP assay buffer, and 50 μL of the supernatant was collected and added to a 96-well plate. Then, 50 μL of ATP assay buffer containing an ATP probe, an ATP converter, and a developer was added to each well. The absorbance was measured at 570 nm.

### Metabolomics

Targeted metabolomic analysis by liquid chromatography–tandem mass spectrometry (LC‒MS/MS) was performed as previously described^16^. Briefly, cells were grown to 60% confluence in growth medium in 10 cm dishes. After 24 h, the cells were washed several times with phosphate-buffered saline and water, harvested using 1.4 mL of cold methanol/H_2_O (80/20, v/v), and lysed by vigorous vortexing. A 100 μL aliquot of a 5 μM internal standard was then added to the cells. Metabolites were obtained by liquid–liquid extraction from the aqueous phase after the addition of chloroform. The aqueous phase was dried using vacuum centrifugation, and the sample was reconstituted with 50 μL of 50% methanol prior to LC‒MS/MS analysis. The LC/MS/MS system was equipped with an Agilent 1290 HPLC instrument (Agilent Technologies, Santa Clara, CA, USA), a QTRAP 5500 (ABSciex, Concord, Ontario, Canada), and a reversed-phase column (Synergi Fusion RP 50 × 2 mm).

### Orthotopic pancreatic tumor mouse model

The pancreases of C57BL/6 mice (6–8 weeks old; Central Lab. Animals, Inc.) were orthotopically injected with cells using an established surgical method. All the experimental procedures were approved by the Institutional Animal Care and Use Committee of the Asan Institute for Life Sciences (2023-12-025). For orthotopic injection, mice were anesthetized under isoflurane gas, and the abdominal skin and muscle were incised just lateral to the midline and directly above the pancreas to allow visualization of the pancreatic lobes. The pancreas was then gently retracted and positioned to allow direct injection of a 30 µL bolus of 1 × 10^6^ Pan02 cells in Matrigel using a 0.3 cc syringe with a 30-gauge needle. The pancreas was then returned to the abdominal cavity, and both the muscle and skin layers were closed with surgical sutures. Following recovery from surgery, the mice were monitored daily. To determine the efficacy of 2DG or BPTES treatment, the mice were divided into six groups (*n* = 6 mice per group) and injected intraperitoneally three times per week with sample buffer (10% DMSO) with or without TRAIL (5 μg/kg/day), 2DG (500 mg/kg/day) with or without TRAIL (5 μg/kg/day), or BPTES (12.5 mg/kg/day) with or without TRAIL (5 μg/kg/day). After harvesting, the length (L) and width (W) of each pancreatic tumor were measured using a caliper, and the tumor volume was calculated as *TV* = (L × W^2^)/2. The tumors were fixed in 4% paraformaldehyde for histopathological and immunohistochemical analyses.

### Xenograft studies

Male NSG mice (6 weeks old, 20 g) were purchased from Joongah Bio. All the experimental procedures were approved by the Institutional Animal Care and Use Committee of the Asan Institute for Life Sciences (protocols 2021-02-340, 2019-02-244). For each animal, 1 × 10^6^ cells were injected into the flank. Once the xenograft tumors for the study of 2DG or BPTES treatment were established (when the tumor volume was ~50–100 mm^3^), the mice were randomly divided into six groups (*n* = 5 mice per group) and treated with saline buffer (10% DMSO), 2DG (500 mg/kg/day) or BPTES (12.5 mg/kg/day), each with or without TRAIL (5 µg/kg/day), via intraperitoneal injection three times a week for 33 days. The length (L) and width (W) of each tumor were measured using a caliper, and the tumor volume (TV) was calculated as *TV* = (L × W^2^)/2. Tumors were harvested and fixed in 10% neutral buffered formalin for histopathological and immunohistochemical analyses. To test the effect of cFLIP gene expression on in vivo tumor growth, 1 × 10^6^ (GFP or cFLIP knockdown) cells were subcutaneously injected into the flanks of mice. When the xenograft volumes were ~50 mm^3^, the mice in all six groups were treated with 100 µL of saline buffer or TRAIL (5 µg/kg/day) by intraperitoneal injection three times a week for 45 days, and tumor growth was monitored as described above.

### Histological and immunohistochemical analyses

For histological analysis, xenograft tumors were fixed with 10% neutral buffered formalin for 24 h. Sections (4 µm thick) of the paraffin-embedded tumors were mounted onto glass slides. The sections were incubated at 60 °C for 1 h, deparaffinized in xylene, and rehydrated through a graded alcohol series. The sections were incubated with anti-cleaved caspase 3 (9661 s, CST), anti-cleaved caspase 8 (9496 s, CST), anti-Ki67 (ab16667, Abcam, Cambridge, UK) and anti-cFLIP (ab8421, Abcam) antibodies overnight at 4 °C, and color was then developed using the Dako Real™ EnVision™ detection system peroxidase/DAB kit (Dako, Glostrup, Denmark). One set of tissue sections was stained with hematoxylin and eosin according to the manufacturer’s instructions. The sections were counterstained with hematoxylin and evaluated by light microscopy (OLYMPUS-cell Sens Standard).

### Flow cytometric analysis of TRAIL receptors

The cell surface expression of cell death receptors was quantified by flow cytometry using the following phycoerythrin-conjugated antibodies: mouse IgG isotype control (12-4714-82, Thermo Fisher), mouse anti-human CD261 (DR4, Biolegend, San Diego, CA, USA), and mouse anti-human CD 262 (DR5, Biolegend). Briefly, cells were harvested with 0.5 mM EDTA and washed with blocking solution (0.5% BSA in PBS). Then, the cells were centrifuged, resuspended in blocking solution, and immunolabeled using antibodies. The cells were incubated with primary antibodies for 2 h at 4 °C in the dark. After washing, the cells were quantified on a flow cytometer (Beckman Coulter). Histograms were generated and the calculated relative mean fluorescence intensity values determined using Flow Jo software (Tree Star, Inc.).

### TRAIL DISC analysis

For analysis of death-inducing signaling complex (DISC) formation, cells were plated in complete medium. The following day, the medium was replaced with Gln-free medium, and the cells were incubated for an additional 24 h. Then, the cells were treated with 500 ng/mL FLAG-tagged TRAIL (ALX-522-003, ENZO, Lausen, Switzerland) in a CO_2_ atmosphere at 37 °C. The cells were washed with cold PBS and lysed in RIPA buffer containing a protease inhibitor cocktail (11836153001, Roche Diagnostics, Basel, Switzerland) for 30 min on ice. The samples were diluted to achieve a protein content of 500 µg, incubated with 30 µL of mouse FLAG-tagged A/G beads (A2220, Sigma‒Aldrich) overnight at 4 °C and then subjected to immunoblot analysis.

### Methylation-specific polymerase chain reaction

Genomic DNA was extracted using the G-DEX™IIc genomic DNA extraction kit (iNtRON). Bisulfite-treated DNA was amplified by polymerase chain reaction (PCR) using two pairs of methylation-specific PCR (MSP) primers designed to target the CpG islands in the promoter of the cFLIP gene. The sequences of the MSP primers used for amplification of methylated DNA were as follows: Me-cFLIP F, TTAGTTTTGTAGGTTTTTTATTTCGG; Me-cFLIP B, AAATTAACCAAAAATTACTTCCGAT. For unmethylated DNA amplification, the following unmethylated MSP primers were used: UM-cFLIP F, TAGTTTTGTAGGTTTTTTATTTTGG; UM-cFLIP B, AAATTAACCAAAAATTACTTCCAAT. The PCR products were visualized by 2% agarose gel electrophoresis. The gels were photographed by using a gel imaging system.

### Combined bisulfite restriction analysis

The DNA methylation status of each individual locus was investigated by combined bisulfite restriction analysis (COBRA) and bisulfite sequencing (BSS). Bisulfite-modified DNA was used as the template for PCR. PCR was performed using gene-specific bisulfite primers designed with MethPrimer software. The primers cFLIP-F (GTTTTTGGAATTTGATTTAGTTTTGTAG) and cFLIP-B (CCAAAATTAACCAAAAATTACTTCC) were designed to flank the CpG islands of the cFLIP gene and contain at least one restriction site for BstU1. The amplified fragments were digested with the BstU1 restriction enzyme. The digested DNA was loaded onto 2% agarose gels and visualized by UV transillumination.

### Chromatin immunoprecipitation assay

Chromatin immunoprecipitation (ChIP) was performed using a Magna ChIP G kit (MGNA0002, Millipore, Billerica, MA, USA) according to the manufacturer’s instructions. For DNA‒protein crosslinking, cells were treated with 1% formaldehyde. The crosslinked chromatin in the cell extracts was sheared by sonication. Chromatin extracts containing DNA fragments were subjected to immunoprecipitation with an anti-H3AC antibody (06-599), an anti-H3K9me3 antibody (3782120) (Millipore), an anti-H3K27me3 antibody (9733) and normal rabbit IgG (2729 s) or mouse IgG as the negative control (sc2025). The human cFLIP-F1 (GAAATCCAGGCTGCAGTGAG), human cFLIP-B1 (GGCGCGAGTGTTTACAATTG), cFLIP-F2 (AGTGGCACGCAGTAGAACAAA), and cFLIP-B2 (GTCCAAAAAGCTTTACTTATGC) primers were designed to flank the CpG islands in the cFLIP gene. Immunoprecipitated DNA was quantified by real-time PCR.

### Statistics

The data are presented as the means ± standard deviations. All comparisons were performed using unpaired Student’s *t*-test.

## Results

### Glutamine is required for TRAIL resistance in PDAC

**Fig. 1:**
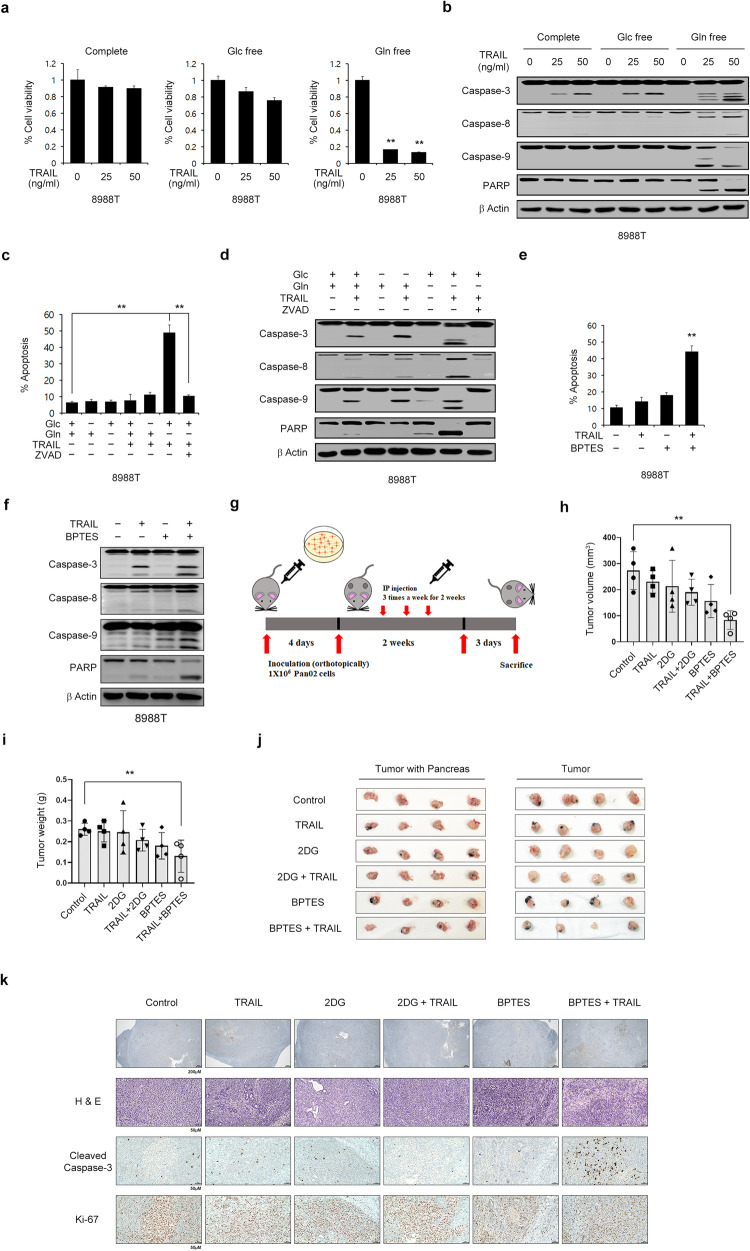
Glutamine deprivation sensitizes PDAC cells to TRAIL-induced apoptosis. **a**, **b** 8988 T cells were plated in complete medium. The next day, the medium was replaced with glucose- or glutamine-free medium; then, the cells were incubated for an additional 24 h, treated with TRAIL at the indicated concentration for 4 h, and subjected to cell viability assays (**a**). Lysates were subjected to immunoblotting with the indicated antibodies (**b**). The error bars indicate the s.d. of triplicate wells from a representative experiment. **c**, **d** 8988 T cells were cultured as described in (**a**) and treated with TRAIL at the indicated concentration for 4 h in the presence or absence of zVAD-fmk (50 μM). Cell death was assessed by Annexin V/PI staining and flow cytometry (**c**), and lysates were subjected to immunoblotting with the indicated antibodies (**d**). The error bars indicate the s.d. of triplicate wells from a representative experiment. **e**, **f** 8988 T cells were treated with BPTES (20 μM) for 24 h and then treated with TRAIL (50 ng/mL) for 4 h. Cell death was assessed by Annexin V/PI staining and flow cytometry (**e**), and lysates were subjected to immunoblotting with the indicated antibodies (**f**). The error bars indicate the s.d. of triplicate wells from a representative experiment. **g** Schematic showing the setup for the animal experiment. Randomized Pan02 cell-bearing mice were treated with 2DG (500 mg/kg/day) or BPTES (12.5 mg/kg/day) with or without TRAIL (5 µg/kg/day) for 14 days. **h**, **i** Tumor volumes and weights were determined on the indicated days. The error bars indicate the s.e.m. values. **j** Representative images of the orthotopic tumor and pancreatic tissue (left) and tumors dissociated from the pancreases (right) harvested from the xenografted mice. **k** Representative sections from each orthotopic xenograft tumor were stained with hematoxylin and eosin (H&E). Representative images of immunohistochemical (IHC) staining with the indicated antibodies are shown for the paired samples. IHC staining was performed using tissues from xenografts of the same passage number used for imaging of H&E staining (scale bars, 50 µm). Glc, glucose; Gln, glutamine. **P < 0.01.

We first examined whether altering a specific nutrient and its metabolism affects apoptosis and could thus be used as a strategy to overcome resistance to TRAIL. To this end, we investigated the effect of depriving cells of glucose or Gln, which are major sources of energy and biosynthesis in proliferating tumor cells^17^, on TRAIL resistance. We found that PDAC cell lines were resistant to TRAIL-induced apoptosis (Fig. 1a, b and Supplementary Fig. 1a, b). Glucose deprivation had no significant effect on PDAC cell viability or apoptosis in the presence of TRAIL, whereas Gln deprivation markedly decreased cell proliferation and significantly activated apoptosis upon TRAIL treatment. (Fig. 1a, b and Supplementary Fig. 1a, b). Treatment with benzyloxycarbonyl-Val-Ala-Asp-(OMe)- fluoromethyl ketone inhibited TRAIL-induced apoptotic cell death under Gln deprivation (Fig. 1c, d and Supplementary Fig. 1c, d).

We next explored the effect of combination treatment with TRAIL and BPTES, an inhibitor of glutaminase, a key enzyme in Gln metabolism, in vitro as well as in vivo in an orthotopic pancreatic tumor mouse model. As shown in Fig. 1e and Supplementary Fig. 1e, BPTES sensitized PDAC cells to TRAIL-induced apoptosis. Consistently, TRAIL activated the initiator caspase-8, the effector caspase-3, and caspase-9 only in the presence of BPTES (Fig. 1f and Supplementary Fig. 1f). We next tested the effect of combination treatment with TRAIL and BPTES in an orthotopic pancreatic tumor mouse model (Fig. 1g). As shown in Fig. 1h–j, tumor growth was significantly inhibited and the tumor weight was markedly reduced after combination treatment with TRAIL and BPTES. Immunohistochemical analysis revealed that TRAIL and BPTES synergistically decreased the tumor weight (Fig. 1k). To further confirm the antitumor effects of combination treatment with TRAIL and BPTES on the survival of human pancreatic cells, we assessed the ability of these cells to grow in vivo as xenografts (Supplementary Fig. 2a). As shown in Supplementary Fig. 2b, c, the strongest inhibition of tumor growth was observed after combination treatment with TRAIL and BPTES. Histological examination of xenograft tumors confirmed the synergistic antitumor effect of TRAIL and BPTES (Supplementary Fig. 2d). Taken together, these results demonstrate that the inhibition of Gln metabolism increases the therapeutic effect of TRAIL, suggesting that this combination could be used to enhance the limited effect of TRAIL for pancreatic cancer therapy.

### Glutamine is critical for maintaining the cFLIP level

**Fig. 2:**
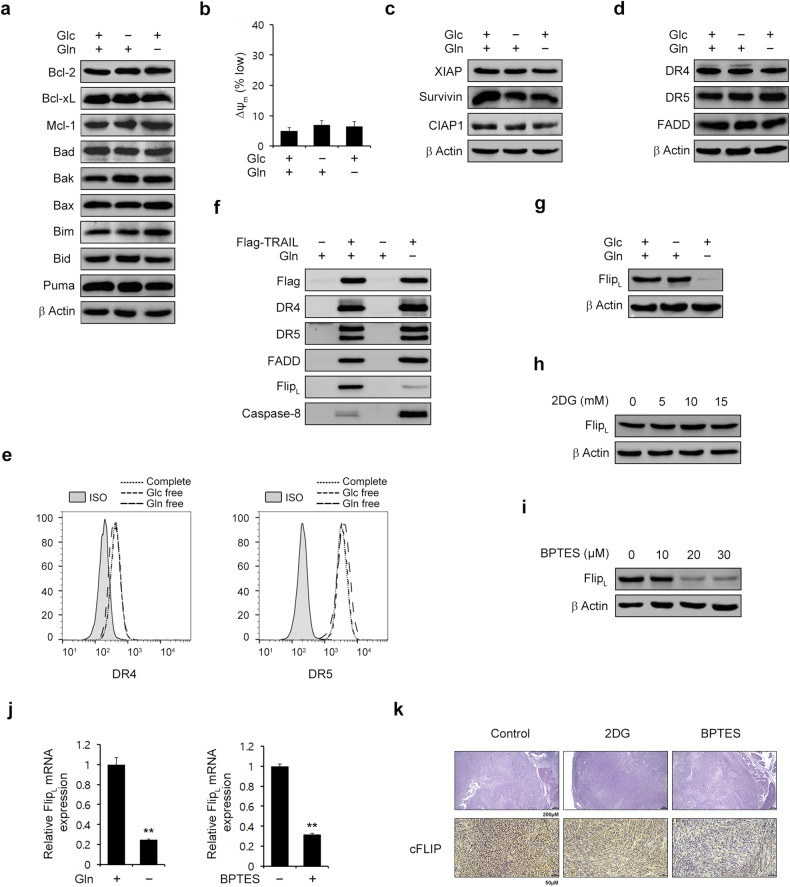
Glutamine deprivation downregulates cFLIP. **a**, **b** 8988 T cells were plated in complete medium; the medium was replaced the following day with glucose- or glutamine-free medium, the cells were incubated for an additional 24 h, and cell lysates were subjected to immunoblotting with the indicated antibodies (**a**). The Δψ_m_ was assessed by flow cytometry using the fluorescent dye TMRE (25 nM) (**b**). The error bars indicate the s.d. of triplicate wells from a representative experiment. **c**, **d** 8988 T cells were plated in complete medium; the medium was replaced the following day with glucose- or glutamine-free medium, the cells were incubated for another 24 h, and cell lysates were subjected to immunoblotting with the indicated antibodies. **e** 8988 T cells were cultured as described in (**a**–**d**) and after an additional 24 h of incubation, the cells were stained with anti-ISO, anti-DR4, and anti-DR5 antibodies. The surface expression of DR4 and DR5 was measured by flow cytometry. **f** 8988 T cells were cultured as described in (**a**–**d**) and treated with Flag-tagged TRAIL (500 ng/mL). Cell lysates were subjected to immunoprecipitation using anti-Flag agarose beads and immunoblotting with the indicated antibodies. **g** 8988 T cells were cultured as described in (**a**–**d**) and the lysates were subjected to immunoblotting with the indicated antibodies. **h** and **i** 8988 T cells were treated with 2DG or BPTES at the indicated concentrations for 24 h, and the lysates were subjected to immunoblotting with the indicated antibodies. **j** cFLIP mRNA expression was measured by quantitative RT‒PCR analysis of 8988 T cells that were plated in complete medium; the medium was replaced the following day with glutamine-free medium, and the cells were incubated for an additional 24 h or treated with BPTES (20 μM) for 24 h. **k** The expression of cFLIP was evaluated in tissue sections from xenograft tumors using IHC staining.

To gain mechanistic insight into the increased TRAIL sensitivity caused by Gln deprivation, we explored the effect of Gln deprivation on the factors involved in the TRAIL-mediated extrinsic pathway of apoptosis. We first measured the expression of BCL-2 family members, which showed that deprivation of glucose or Gln did not affect the expression of BCL-2 family members (Fig. 2a). Consistent with this result, nutrient deprivation had no effect on the mitochondrial membrane potential (Fig. 2b) or on the levels of inhibitors of apoptosis (Fig. 2c). Because death receptors have been suggested to be targets for TRAIL sensitization^18–20^, we tested the effect of nutrient deprivation on death receptor expression. Nutrient deprivation had no effect on the intracellular expression of death receptors (Fig. 2d) or the cell surface expression of death receptors in PDAC cells (Fig. 2e). Because the formation of the DISC plays an important role in TRAIL-induced apoptosis, we next performed DISC analysis and found that Gln deprivation significantly decreased the recruitment of cFLIP to the DISC and promoted the recruitment of caspase-8 to the DISC (Fig. 2f). Gln deprivation but not glucose deprivation significantly decreased the cFLIP protein level (Fig. 2g). Consistent with this result, 2DG treatment had no effect on the cFLIP level, whereas BPTES treatment decreased the cFLIP level in a dose-dependent manner (Fig. 2h, i). In addition, both Gln deprivation and BPTES treatment dramatically reduced the cFLIP mRNA level (Fig. 2j). Immunohistochemical analysis revealed that BPTES treatment decreased the expression of cFLIP in xenograft tumors (Fig. 2k and Supplementary Fig. 2e). It has been reported that the increased ATF4 level following Gln deprivation exerts critical effects on TRAIL-induced apoptosis by regulating apoptotic factors such as death receptor (DR) 5 in breast cancer^19^. Thus, we tested the effect of ATF4 on the glutamine deprivation-mediated apoptosis pathway. As shown in Supplementary Fig. 3a, glutamine deprivation significantly increased both the ATF4 and CHOP levels, consistent with the role of ATF4 as a stress-responsive transcription factor. We also observed that ATF4 knockdown not only failed to rescue cells from glutamine deprivation-mediated TRAIL-induced apoptosis but instead led to increased apoptosis (Supplementary Fig. 3b). In addition, overexpression of ATF4 had no significant effect on TRAIL-induced apoptosis (Supplementary Fig. 3c). Overexpression of ATF4 did not affect the expression of BCL-2 family members (Supplementary Fig. 3d). Additionally, it had no effect on the expression levels of inhibitors of apoptosis (Supplementary Fig. 3e) or on the cellular expression of death receptors (Supplementary Fig. 3f), suggesting that the role of ATF4 in glutamine deprivation-mediated apoptosis is complex and dependent on the tumor type. Taken together, these data indicate that Gln deprivation downregulates cFLIP, leading to the recruitment of caspase-8 to the DISC, and that its activation is mediated through a mechanism different from that previously reported.

### cFLIP is indispensable for glutamine deprivation-mediated TRAIL sensitization

**Fig. 3:**
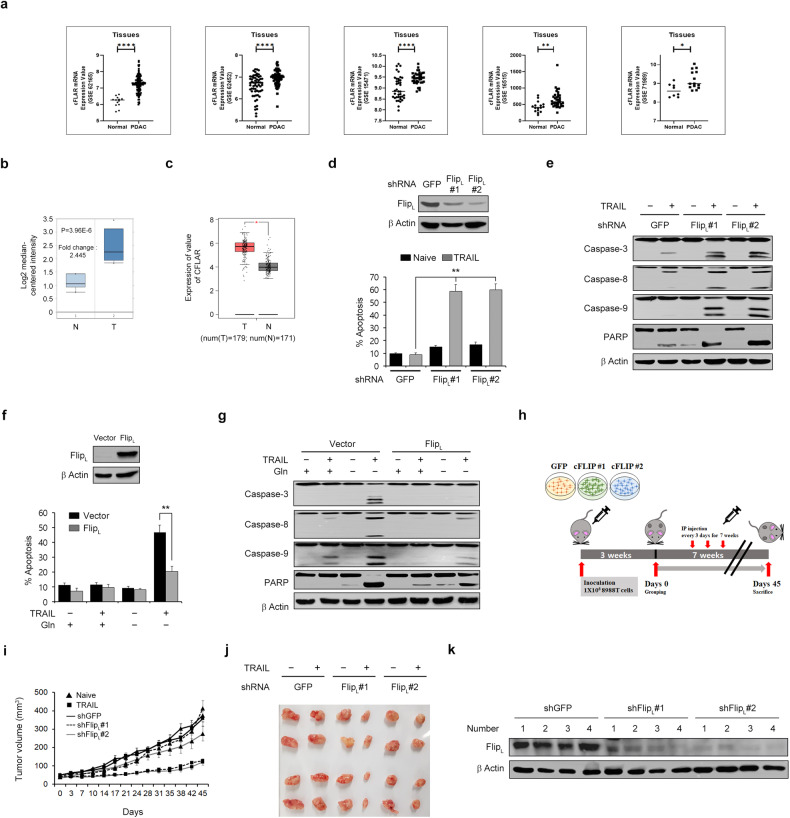
cFLIP is required for sensitivity to TRAIL-induced apoptosis in PDAC cells. **a** The cFLIP level was compared between normal pancreatic tissues and PDAC tissues. (**b**) and (**c**) Box plots generated from gene expression data in Oncomine comparing the mRNA expression level of cFLIP between normal tissue samples (left plot) and pancreatic cancer samples (right plot) (**b**). The mRNA expression level of cFLIP was measured in 179 pancreatic cancer tissues (T) and 171 normal tissues (N) represented in the GEPIA database (**c**). (**d**) and (**e**) 8988 T cells expressing control shRNA (shGFP) or shRNAs targeting cFLIP were treated with TRAIL (50 ng/mL) for 4 h, and cell death was assessed by Annexin V/PI staining and flow cytometry (**d**). Lysates were subjected to immunoblotting with the indicated antibodies (**e**). The error bars indicate the s.d. of triplicate wells from a representative experiment. **f** and **g** 8988 T cells expressing pCDH (empty vector) or pCDH-FLIP_L_ (cFLIP) were plated in complete medium. The next day, the medium was replaced with glutamine-free medium, the cells were incubated for an additional 24 h and treated with TRAIL (50 ng/mL) for 4 h, and cell death was assessed by Annexin V/PI staining and flow cytometry (**f**). Lysates were subjected to immunoblotting with the indicated antibodies (**g**). The error bars indicate the s.d. of triplicate wells from a representative experiment. **h** Schematic showing the setup for the animal experiment. 8988 T cells expressing control shRNA (shGFP) or shRNAs targeting cFLIP were injected into mice, and the mice were treated with or without TRAIL (5 µg/kg/day) for 45 days. **i** Tumor volumes were calculated on the indicated days. The error bars indicate the s.e.m. values. **j** Representative images of the xenograft tumors obtained from the mice. **k** Xenograft tumors were collected, and tissue lysates were subjected to immunoblot analysis of cFLIP expression. *P < 0.05, **P < 0.01. ***P < 0.001.

Given that cFLIP is downregulated upon inhibition of Gln metabolism, we speculated that cFLIP expression might be elevated in PDAC cells and act as a regulator of TRAIL resistance. First, we evaluated the expression of cFLIP using data from The Cancer Genome Atlas (TCGA) obtained via the Gene Expression Omnibus repository (GSE62165, GSE62452, GSE15471, GSE16515, and GSE71989). As shown in Fig. 3a, the expression of cFLIP was significantly higher in PDAC tumors than in normal tissues. Consistently, analysis of TCGA data for pancreatic cancer patients using GEPIA and Oncomine showed that the cFLIP level is highly elevated in PDAC tumors relative to normal tissues (Fig. 3b, c), indicating that the increased cFLIP level may be involved in TRAIL resistance in PDAC cells.

To further assess the role of cFLIP in TRAIL resistance, we knocked down cFLIP, which significantly induced apoptotic cell death following TRAIL treatment (Fig. 3d). Consistently, cFLIP knockdown activated caspase-3, caspase-8, caspase-9, and PARP upon TRAIL treatment (Fig. 3e). To confirm that cFLIP acts as a key regulator of Gln deprivation-mediated TRAIL sensitization, we examined the effect of cFLIP overexpression on apoptosis. As shown in Fig. 3f, overexpression of cFLIP significantly decreased the cell death caused by TRAIL treatment under conditions of Gln deprivation. cFLIP overexpression also inhibited the TRAIL-induced cleavage of caspase-3, caspase-8, caspase-9, and PARP under Gln deprivation conditions (Fig. 3g). We next examined the effect of TRAIL combined with downregulation of cFLIP in a xenograft mouse model (Fig. 3h). cFLIP knockdown significantly inhibited tumor growth following TRAIL treatment (Fig. 3i). The size of the xenograft tumors was dramatically decreased in response to combined TRAIL treatment and cFLIP downregulation (Fig. 3j). The efficacy of cFLIP downregulation was confirmed, as shown in Fig. 3k. Taken together, these results indicate that cFLIP is an important factor in Gln deprivation-mediated TRAIL sensitization.

### Glutamine-derived α-ketoglutarate production is required to maintain the cFLIP level

**Fig. 4:**
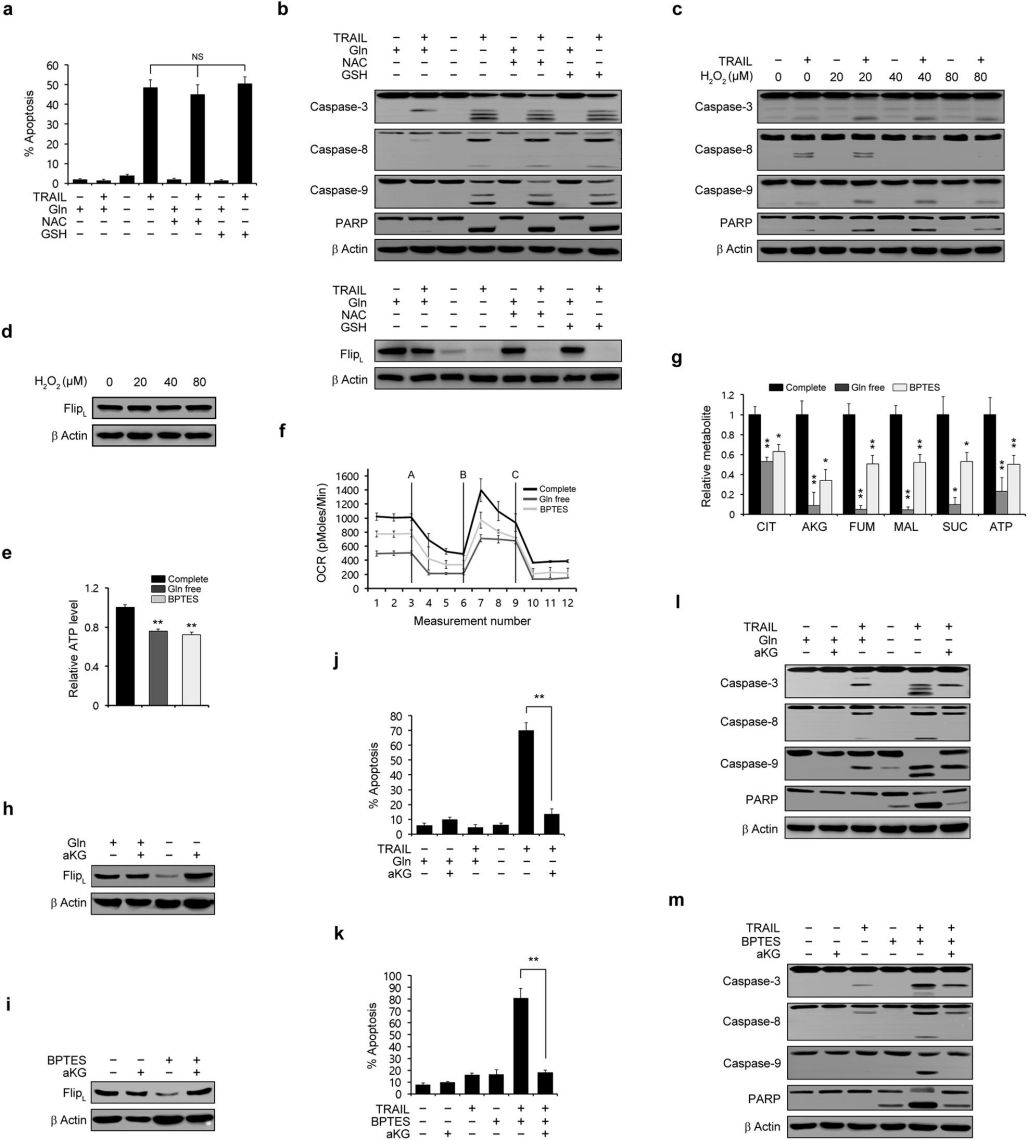
Glutamine-derived aKG is required for sensitivity to TRAIL-induced apoptosis through regulation of cFLIP expression in PDAC cells. (**a**) and (**b**) 8988 T cells were plated in complete medium. The next day, the medium was replaced with glutamine-free medium supplemented with NAC (3 mM) or GSH (3 mM), and the cells were incubated for an additional 24 h prior to treatment with TRAIL (50 ng/mL) for 4 h and assessment of cell death by Annexin V/PI staining and flow cytometry (**a**). Lysates were subjected to immunoblotting with the indicated antibodies (**b**). The error bars indicate the s.d. of triplicate wells from a representative experiment. **c** and **d** 8988 T cells were treated with H_2_O_2_ at the indicated concentrations for 24 h, treated with TRAIL (50 ng/mL) for 4 h, and the lysates were subjected to immunoblotting with the indicated antibodies. **e**–**g** 8988 T cells were plated in complete medium. The next day, the medium was replaced with glutamine-free medium or treated with BPTES (10 μM) for 24 h. The intracellular ATP level was measured (**e**), and the oxygen consumption rate was measured using an extracellular flux analyzer (**f**). The TCA metabolite pools in the cells were analyzed by LC‒MS/MS (**g**). The error bars indicate the s.d. of triplicate wells from a representative experiment. **h** 8988 T cells were plated in complete medium. The next day, the medium was replaced with glutamine-free medium supplemented with aKG (4 mM) for 24 h, and the cell lysates were subjected to immunoblotting with the indicated antibodies. **i** 8988 T cells were treated with BPTES (20 μM) for 24 h in the presence or absence of aKG (4 mM), and the lysates were subjected to immunoblotting with the indicated antibodies. **j** and **l** 8988 T cells were plated in complete medium. The next day, the medium was replaced with glutamine-free medium supplemented with aKG (4 mM) for 24 h, and the cells were treated with TRAIL (50 ng/mL) for 4 h. Cell death was assessed by Annexin V/PI staining and flow cytometry (**j**), and the lysates were subjected to immunoblotting with the indicated antibodies (**l**). The error bars indicate the s.d. of triplicate wells from a representative experiment. **k** and **m** 8988 T cells were treated with BPTES (20 μM) for 24 h in the presence or absence of aKG (4 mM) and then treated with TRAIL (50 ng/mL) for 4 h. Cell death was assessed by Annexin V/PI staining and flow cytometry (**k**), and the lysates were subjected to immunoblotting with the indicated antibodies (m). The error bars indicate the s.d. of triplicate wells from a representative experiment. NS, not significant. * *P* < 0.05, ** *P* < 0.01.

ROS are involved in the degradation of the cFLIP protein^21–24^. Gln is essential for maintaining redox homeostasis and supporting the growth of PDAC cells^25^. Thus, we tested whether the TRAIL-mediated apoptotic cell death induced under Gln deprivation conditions is due to ROS-mediated cFLIP downregulation. Treatment with *N*-acetylcysteine (NAC) or glutathione (GSH) did not rescue cells from apoptotic cell death induced by TRAIL treatment under Gln deprivation conditions (Fig. 4a). Additionally, it did not inhibit the cleavage of caspase-3, caspase-8, caspase-9, or PARP under these conditions, nor did it reverse the reduction in the cFLIP level caused by Gln deprivation (Fig. 4b). Additionally, hydrogen peroxide (H_2_O_2_) treatment did not sensitize PDAC cells to TRAIL-induced apoptosis (Fig. 4c), and the level of cFLIP was not altered following H_2_O_2_ treatment (Fig. 4d), indicating that Gln deprivation-mediated TRAIL sensitization is not related to the maintenance of ROS levels.

Gln is a carbon source for the TCA cycle, and blocking Gln metabolism impairs tumor growth^26,27^. Therefore, we first investigated the role of Gln as a fuel source for the TCA cycle to determine the involvement of Gln in regulating cFLIP expression. As shown in Fig. 4e, f, Gln deprivation or BPTES treatment significantly decreased the ATP level (Fig. 4e) and oxygen consumption rate (Fig. 4f). The effect of Gln deprivation on the abundances of TCA cycle intermediates was explored by metabolomic analysis, which showed that Gln deprivation or BPTES treatment significantly decreased the abundances of TCA cycle intermediates (Fig. 4g), indicating that Gln is a fuel source for the TCA cycle in PDAC cells. To determine whether the downregulation of cFLIP upon Gln deprivation is caused by an insufficient supply of nutrients for the TCA cycle, we examined the effect of supplementation with aKG, which provides substrates for the TCA cycle, on the cFLIP level. As shown in Fig. 4h, i, aKG supplementation reversed the reduction in the cFLIP level induced by either Gln deprivation or BPTES treatment. AKG supplementation dramatically inhibited apoptotic cell death caused by TRAIL treatment upon inhibition of Gln metabolism under Gln deprivation conditions or BPTES treatment (Fig. 4j, k). Consistently, aKG supplementation inhibited caspase-3, caspase-8, caspase-9, and PARP cleavage induced by TRAIL upon Gln deprivation or BPTES treatment (Fig. 4l, m). Therefore, these results demonstrate that maintenance of the cFLIP level requires Gln-derived aKG production.

### GOT2-mediated aKG production is critical for maintaining the cFLIP level

**Fig. 5:**
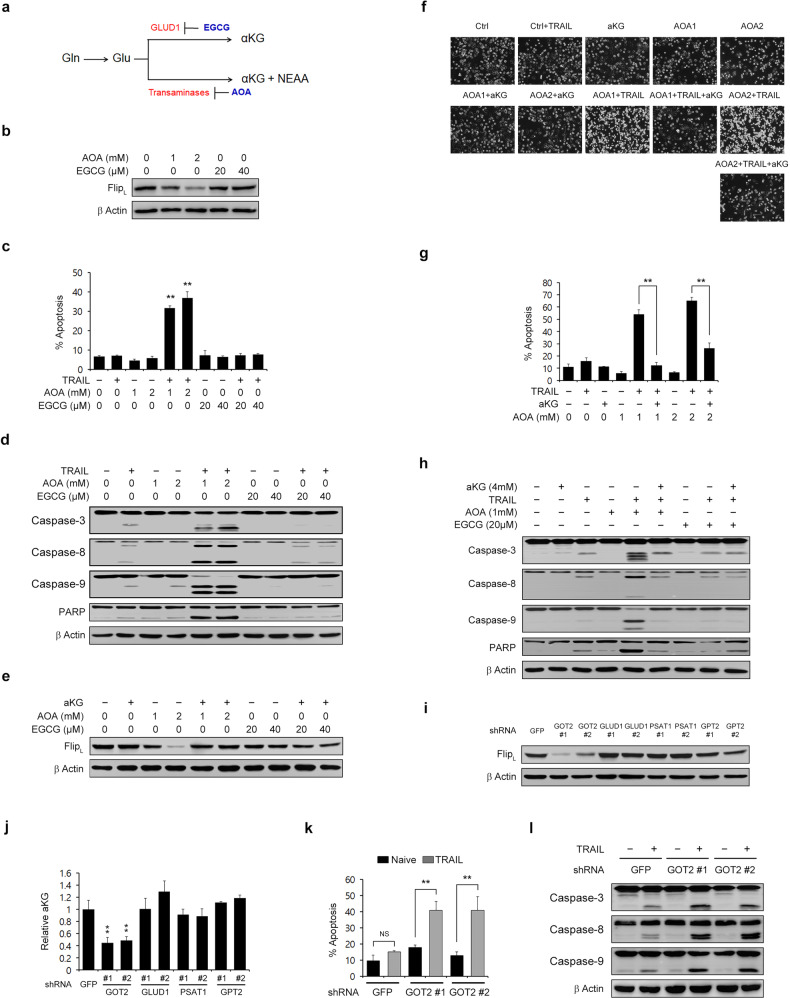
The mitochondrial transaminase GOT2 is required for the regulation of cFLIP expression upon the activation of TRAIL-induced apoptosis. **a** Schematic showing the reaction by which glutamate (Glu) is converted to aKG. **b** 8988 T cells were treated with AOA or EGCG at the indicated concentrations for 24 h, and the lysates were subjected to immunoblotting with the indicated antibodies. **c** and **d** 8988 T cells were treated with AOA or EGCG at the indicated concentrations for 24 h and treated with TRAIL (50 ng/mL) for 4 h. Cell death was assessed by Annexin V/PI staining and flow cytometry (**c**), and the lysates were subjected to immunoblotting with the indicated antibodies (**d**). The error bars indicate the s.d. of triplicate wells from a representative experiment. **e** 8988 T cells were treated with AOA or EGCG at the indicated concentrations for 24 h in the presence or absence of aKG (4 mM), and the lysates were subjected to immunoblotting with the indicated antibodies. **f** and **g** 8988 T cells were treated with AOA at the indicated concentrations for 24 h in the presence or absence of aKG (4 mM) and then treated with TRAIL (50 ng/mL). Cell morphology was analyzed under an inverted microscope (magnification, 100×) (**f**), and cell death was assessed by Annexin V/PI staining and flow cytometry (**g**). The error bars indicate the s.d. of triplicate wells from a representative experiment. **h** 8988 T cells were treated with AOA or EGCG at the indicated concentrations for 24 h in the presence or absence of aKG (4 mM), treated with TRAIL (50 ng/mL) for 4 h and then subjected to immunoblotting with the indicated antibodies. **i** 8988 T cells expressing control shRNA (GFP), GOT2 shRNAs, GLUD1 shRNAs, PSAT1 shRNAs, or GPT2 shRNAs were subjected to immunoblotting with the indicated antibodies. **j** AKG metabolite abundances in 8988 T cells expressing control shRNA (GFP), GOT2 shRNAs, GLUD1 shRNAs, PSAT1 shRNAs, or GPT2 shRNAs. The error bars indicate the s.d. of triplicate wells from a representative experiment. **k** and **l** 8988 T cells expressing control shRNA (GFP) or GOT2 shRNAs were treated with TRAIL (50 ng/mL), and cell death was assessed by Annexin V/PI staining and flow cytometry (**k**). Lysates were subjected to immunoblotting with the indicated antibodies (**l**). The error bars indicate the s.d. of triplicate wells from a representative experiment. **P < 0.01.

Gln is first converted into glutamate (Glu) by glutaminase, which is localized in mitochondria^28^, and mitochondrial Glu is subsequently converted into aKG by Glu dehydrogenase 1 (GLUD1) or by transaminases, including glutamic-oxaloacetic transaminase 2 (GOT2)^29^ (Fig. 5a). We first tested which metabolic pathway is critical for Gln-mediated cFLIP regulation. Epigallocatechin gallate (EGCG), an inhibitor of GLUD1, had no effect on the cFLIP level, whereas aminooxyacetate (AOA), a pan-inhibitor of transaminases, robustly reduced the cFLIP level (Fig. 5b). To confirm the importance of transaminases in Gln deprivation-mediated TRAIL sensitization, we examined the effect of treatment with either AOA or EGCG on apoptotic cell death following TRAIL treatment. EGCG did not activate apoptosis when combined with TRAIL, whereas the combination of TRAIL with AOA significantly increased apoptosis (Fig. 5c and Supplementary Fig. 4a). Consistent with this result, the combination of TRAIL with AOA but not with EGCG induced caspase-3, caspase-8, caspase-9, and PARP cleavage (Fig. 5d and Supplementary Fig. 4b). AKG supplementation significantly reversed the decrease in the cFLIP level induced by AOA treatment (Fig. 5e) and restored the morphology of PDAC cells, which was altered by combination treatment with TRAIL and AOA (Fig. 5f). In addition, aKG supplementation significantly rescued cells from apoptosis caused by combination treatment with TRAIL and AOA (Fig. 5g and Supplementary Fig. 4c) and inhibited caspase-3, −8, and −9 and PARP cleavage caused by this combination treatment (Fig. 5h and Supplementary Fig. 4d). To identify the specific transaminase involved in cFLIP regulation, Glu-dependent transaminases (aspartate, alanine, and phosphoserine transaminases) were inhibited by the introduction of specific RNAi constructs, and the level of cFLIP was measured. Knockdown of only GOT2 (mitochondrial form), among the Glu-dependent transaminases, dramatically reduced the cFLIP level (Supplementary Fig. 5 and Fig. 5i). Metabolomic analysis revealed that GOT2 knockdown decreased the aKG level (Fig. 5j and Supplementary Fig. 4e). GOT2 knockdown significantly increased apoptosis (Fig. 5k and Supplementary Fig. 4f) and caspase-3, −8, and −9 cleavage following TRAIL treatment (Fig. 5l and Supplementary Fig. 4g). Knockdown of GOT1 (cytoplasmic form) had no effect on the cFLIP level (Supplementary Fig. 6a), apoptotic cell death, or the cleavage of caspase-3, −8, or −9 when combined with TRAIL treatment (Supplementary Fig. 6b, c). Taken together, these results suggest that the maintenance of the cFLIP level requires GOT2-derived aKG production.

### Gln-derived aKG is essential for KDM4C-mediated epigenetic regulation of cFLIP

**Fig. 6:**
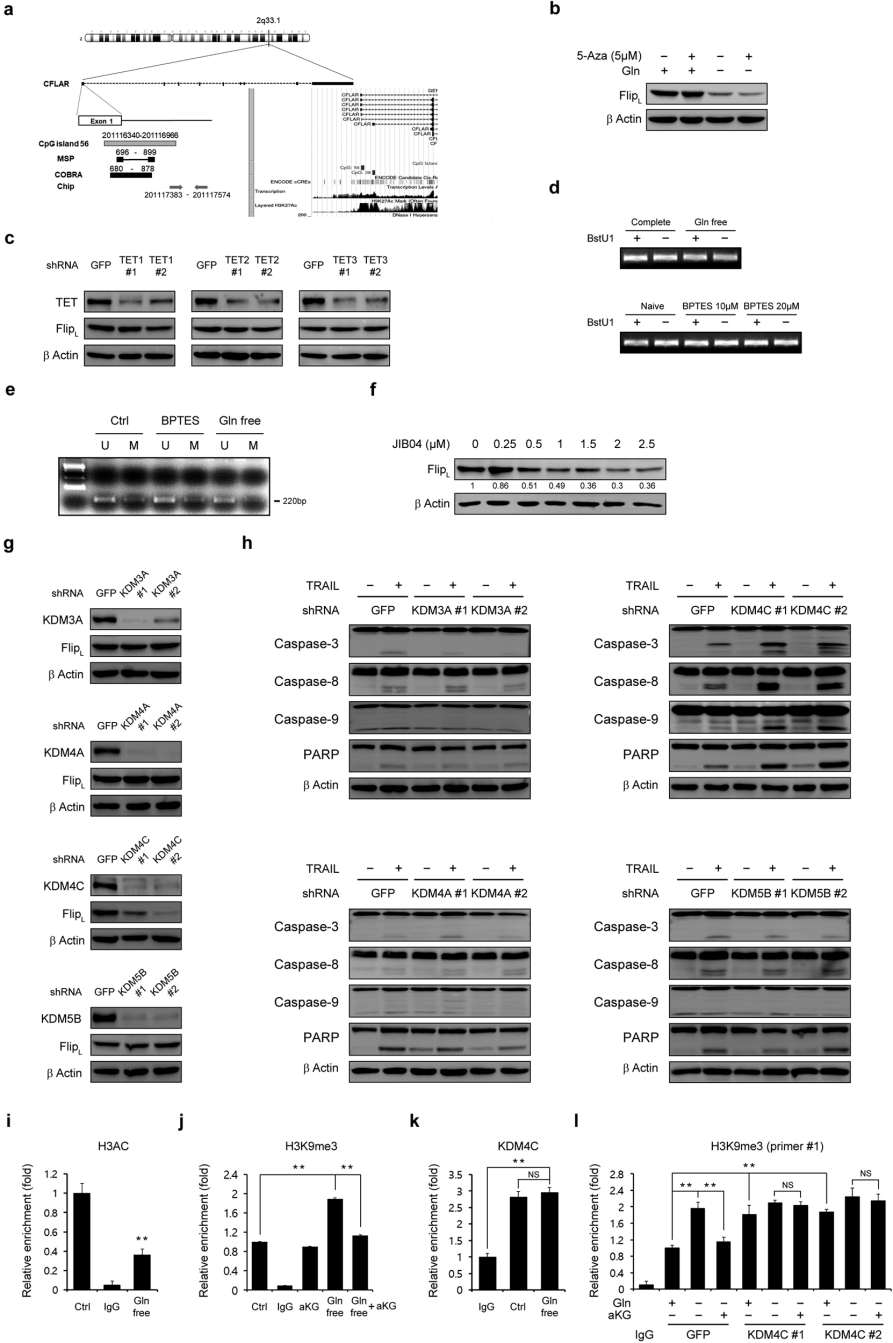
Glutamine-derived aKG is required for epigenetic regulation of cFLIP. **a** Map of CpG islands in the cFLIP promoter showing the regions targeted by the primers used for MSP, COBRA, and ChIP. **b** 8988 T cells were plated in complete medium. The next day, the medium was replaced with glutamine-free medium, and the cells were treated with or without 5-Aza (5 μM) for 24 h and subjected to immunoblotting with the indicated antibodies. **c** 8988 T cells expressing control shRNA (GFP) or TET isoform shRNAs were subjected to immunoblotting with the indicated antibodies. **d** 8988 T cells were plated in complete medium. The next day, the medium was replaced with glutamine-free medium, and the cells were incubated for an additional 24 h or treated with BPTES for 24 h prior to analysis of DNA methylation by MSP. **e** 8988 T cells were cultured as described in (**d**) and incubated for an additional 24 h or treated with BPTES (20 μM) for 24 h prior to analysis of DNA methylation using COBRA. **f** 8988 T cells were treated with JIB04 at the indicated concentrations for 24 h, and the lysates were subjected to immunoblotting with the indicated antibodies. **g** 8988 T cells expressing control shRNA (GFP), KDM3A shRNAs, KDM4A shRNAs, KDM4C shRNAs, or KDM5B shRNAs were subjected to immunoblotting with the indicated antibodies. **h** 8988 T cells expressing control shRNA (GFP), KDM3A shRNAs, KDM4A shRNAs, KDM4C shRNAs, or KDM5B shRNAs were treated with TRAIL (50 ng/mL) for 4 h, and the lysates were subjected to immunoblotting with the indicated antibodies. **i** 8988 T cells were cultured as described in (**d**) and incubated for an additional 24 h. Chromatin was prepared for analysis of H3AC. The error bars indicate the s.d. of triplicate wells from a representative experiment. **j** 8988 T cells were plated in complete medium; the medium was replaced the following day with glutamine-free medium supplemented with aKG (4 mM), and the cells were incubated for an additional 24 h. Chromatin was prepared for analysis of H3K9me3. The error bars indicate the s.d. of triplicate wells from a representative experiment. **k** 8988 T cells were plated in complete medium. The next day, the medium was replaced with glutamine-free medium, and the cells were incubated for an additional 24 h. Chromatin was prepared for analysis of KDM4C activity. **l** Chromatin was prepared to examine the methylation status of H3K9me3 in 8988 T cells expressing control shRNA (GFP) or KDM4C shRNAs. ChIP was performed with comparison to isotype control (Ctrl). The ChIP eluates were subjected to Q-PCR analysis of the indicated regions of the cFLIP gene locus. The error bars indicate the s.d. of triplicate wells from a representative experiment. **P < 0.01.

The above data indicate that cFLIP downregulation caused by Gln deprivation leads to TRAIL-induced apoptosis in PDAC cells and that aKG supplementation reverses the reduction in the cFLIP level induced by Gln deprivation and rescues PDAC cells from TRAIL-induced apoptosis. These results suggest that the downregulation of cFLIP upon Gln deprivation involves aKG-mediated epigenetic regulation. Metabolite profiles play a critical role in the epigenetic regulation of gene expression by modulating the activity of DNA and histone modification enzymes. In particular, both ten-eleven translocation proteins (TETs) and JmjC domain-containing histone demethylases (JMHDs), which are key players in DNA and histone methylation, are aKG-dependent dioxygenases^30,31^. Therefore, we next investigated the importance of aKG-dependent DNA or histone methylation on cFLIP expression. To analyze the DNA and histone methylation status of the promoter at the cFLIP locus, we performed MSP, COBRA, and ChIP. Figure 6a shows the CpG islands in the cFLIP promoter with the regions targeted by the primers used for MSP, COBRA, and ChIP. First, we examined the effect of DNA methylation on cFLIP expression. As shown in Fig. 6b, treatment with 5-aza-2’-deoxycytidine (5-aza), a DNMT inhibitor, did not restore the decrease in the cFLIP level induced by Gln deprivation. Moreover, knockdown of TETs had no significant effect on the cFLIP level (Fig. 6c). To further determine whether the downregulation of cFLIP induced by the reduction in Gln-derived aKG production is regulated by DNA methylation, we performed COBRA. Neither Gln deprivation nor BPTES treatment altered the methylation status (Fig. 6d), and the cFLIP gene remained unmethylated (Fig. 6e). These results indicate that the downregulation of cFLIP caused by reduced Gln-derived aKG production may not be regulated by DNA methylation. Because JHDM is an epigenetic enzyme that uses aKG as a cosubstrate^32^, we speculated that cFLIP expression may be regulated by aKG-dependent histone modification. We thus examined the effect of JIB04, a pan-JHDM inhibitor^33^, on the cFLIP level. Treatment with JIB04 decreased the cFLIP level in a dose-dependent manner (Fig. 6f). The mechanism underlying cFLIP downregulation in response to decreased Gln-derived aKG production was examined by individual inhibition of a panel of histone lysine demethylases (KDMs) (KDM3A, 4 A, 4 C, and 5B), which have been reported to be the most sensitive to JIB04, using RNAi^33–36^. Knockdown of KDM4C significantly reduced the level of cFLIP (Fig. 6g) and the cleavage of caspase-3, caspase-8, caspase-9, and PARP when combined with TRAIL treatment (Fig. 6h). Consistently, knockdown of only KDM4C significantly increased apoptosis (Supplementary Fig. 7). A ChIP assay was performed to assess histone H3 modification in the cFLIP promoter. As shown in Fig. 6i, Gln deprivation decreased H3AC. The suppression of cFLIP expression following knockdown of KDM4C suggested that this occurred because KDM4C was no longer available to remove methylation marks from H3K9 or H3K27, which are known to repress transcription. To test this hypothesis, we next examined the methylation level of histone H3 at lysine residues K9 and K27. Gln deprivation increased only H3K9me3 (Supplementary Fig. 8a), and aKG supplementation inhibited the increase in H3K9me3 induced by Gln deprivation (Fig. 6j). KDM4C bound directly to the cFLIP promoter, and Gln deprivation did not inhibit KDM4C binding to the cFLIP promoter, indicating that Gln deprivation inhibits the activation of KDM4C-mediated cFLIP transcription by reducing the level of aKG, which is required for KDM4C activity (Fig. 6k). In addition, KDM4C knockdown led to a significant increase in H3K9me3, and this increase in H3K9me3 was not significantly reversed upon aKG supplementation, indicating that KDM4C is required for the Gln-mediated epigenetic regulation of cFLIP (Fig. 6l and Supplementary Fig. 8b). Taken together, these results suggest that Gln-derived aKG is an important cofactor required for histone modification and that decreased Gln-derived aKG production suppresses histone demethylation, specifically H3K9me3 demethylation, by inhibiting KDM4C activity, leading to significant downregulation of cFLIP expression.

## Discussion

**Fig. 7:**
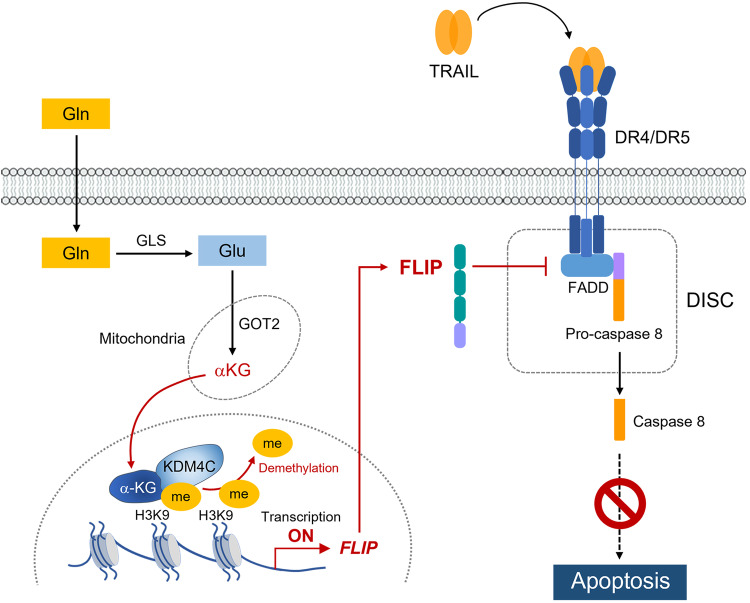
Model depicting glutamine-mediated aKG production in PDAC cells and its role in the epigenetic regulation of cFLIP transcription.

In this study, we elucidated a novel molecular mechanism of resistance to TRAIL in PDAC. Gln plays a critical role in resistance to TRAIL by maintaining the cFLIP level. For the first time, we showed that the histone lysine demethylase KDM4C binds to the cFLIP promoter and epigenetically regulates the expression of cFLIP. PDAC utilizes Gln to produce aKG through GOT2, and Gln-derived aKG is a cofactor required for KDM4C-mediated cFLIP regulation (Fig. 7).

The role of Gln in resistance to TRAIL has been demonstrated in many cancers. Targeting Gln metabolism sensitizes melanoma cells to TRAIL-induced apoptosis by regulating BCL-2 family members^37^. Gln deprivation upregulates death receptors, leading to apoptosis when Gln deprivation is combined with TRAIL treatment^18,38^. AOA, a pan-inhibitor of transaminases, increases the TRAIL-R2 levels and decreases the cFLIP level, thereby promoting apoptosis upon TRAIL treatment^19,20^. However, the detailed molecular mechanisms by which Gln deprivation reverses TRAIL resistance remain unclear. In this study, we clearly showed that Gln deprivation decreased GOT2-mediated aKG production and that the reduced aKG level inhibited KDM4C-mediated cFLIP expression, thereby sensitizing PDAC cells to TRAIL-induced apoptosis.

In cancer, metabolic and epigenetic alterations are highly intertwined because metabolites act as cofactors for epigenetic enzymes. Cancer cell proliferation requires Gln as a source of nitrogen and anaplerosis (the maintenance of TCA cycle intermediates). Although the TCA cycle plays an essential role in ATP production, it is also a source of biosynthetic precursors and chemical intermediates^39^. AKG, a TCA cycle metabolite, is a required cofactor for the histone demethylase JHDM and the DNA demethylase TET, and a reduction in Gln-dependent aKG production inhibits the activity of histone and DNA demethylases, which regulate numerous cancer-related genes^40,41^. Gln-derived glutaminolysis mediates aKG production in many cancers^42^. In particular, PDAC cells require Gln, and supplementation of aKG with nonessential amino acids (NEAAs) rescues PDAC cells from Gln deprivation-mediated growth inhibition^11,26^, supporting the present data showing that Gln is a critical source for aKG production. A study showed that knockdown of GOT1 (cytoplasmic form) but not GOT2 sensitized breast cancer cells to TRAIL-induced apoptosis^19^, inconsistent with the present data. The difference in the results may be due to the utilization of a noncanonical pathway of Gln metabolism in PDAC cells. Kimmelman et al. reported that GOT1- and GOT2-mediated metabolic pathways support PDAC growth by maintaining redox homeostasis^11^. GOT2 catalyzes the conversion of oxaloacetate and glutamate into aspartate and aKG in mitochondria, and GOT1 catalyzes the conversion of aspartate and aKG into oxaloacetate and glutamate in the cytoplasm. The authors of the study referenced above showed that GOT1 knockdown caused the accumulation of Gln-derived aKG, indicating that GOT1 may not be important for the production of aKG in PDAC cells. These data support our results showing that GOT2 is critical for aKG production in PDAC cells.

Many studies have shown that Gln metabolism is required for PDAC growth^11,29^. Consistent with these findings, our data also showed that Gln deprivation led to alterations in metabolic activity, such as a decreased ATP level, which in turn impaired PDAC growth. However, our data showed that Gln deprivation did not induce apoptotic cell death within a short period of time, such as 24 h. There are two different mechanisms by which Gln deprivation activates apoptosis. First, apoptosis is triggered only by Gln deprivation in the absence of apoptotic stimuli such as TRAIL. Second, Gln deprivation sensitizes cancer cells to apoptotic stimuli. Many studies have reported that Gln deprivation induces apoptotic death in cancer cells in the absence of apoptotic stimuli^29^. However, there have been no reports suggesting that apoptotic cell death is induced by Gln deprivation in the absence of apoptotic stimuli in PDAC. In addition, consistent with our data, many studies have reported that Gln deprivation suppresses cancer cell growth and metabolic activity but does not induce apoptosis^19,43,44^. Senescence may promote survival in the absence of Gln because senescent cells are not immediately eliminated and remain metabolically active despite being in a state of growth arrest, and they remain viable with changes in metabolic activity and are usually resistant to apoptosis. However, further work must be performed to investigate the mechanisms underlying the increased survival capacity of PDAC cells during metabolic alterations.

cFLIP overexpression is an important factor in the resistance of cancer cells to death receptor ligands, including TRAIL. cFLIP expression is significantly higher in PDAC tumors than in normal tissues, and cFLIP knockdown increases death receptor-induced apoptosis in PDAC cells^45^, consistent with the present data and suggesting that cFLIP is an effective target for decreasing resistance to TRAIL in PDAC cells. cFLIP expression in cancer cells is regulated at both the transcriptional and posttranslational levels. For example, cFLIP expression is upregulated by NF-κB, p53, p63, NFAT, EGR1, hnRNP K, AR, and sp1 and downregulated by c-myc, Foxo3a, c-Fos, IRF5, and sp3; cFLIP is also ubiquitinated and targeted for proteasome-mediated degradation^46,47^. However, the epigenetic regulation of cFLIP expression remains uncharacterized. In this study, we show, for the first time, that cFLIP expression is epigenetically regulated by the histone demethylase KDM4C. The present study also demonstrates a novel dependence of PDAC cells on Gln for TRAIL resistance. Gln deprivation suppresses GOT2-mediated aKG production, and the decreased aKG level results in inhibition of the KDM4C-mediated promotion of cFLIP transcription, which increases the sensitivity of PDAC cells to TRAIL. Therefore, the present findings may have implications for the design of therapeutic approaches because inhibition of Gln metabolism may act synergistically with TRAIL treatment in PDAC.

### Supplementary information

Supplementary Information
