# Supplementary material for: Glutamine-mediated epigenetic regulation of cFLIP underlies resistance to TRAIL in pancreatic cancer
Source: Exp Mol Med. 2024 Apr 30;56(4):1013–26. doi: 10.1038/s12276-024-01231-0 (PMC11058808; doi:10.1038/s12276-024-01231-0)
Supplement: Supplementary file 1 — Supplementary Information [file 12276_2024_1231_MOESM1_ESM.docx]

**Supplementary Information**

**Glutamine-mediated epigenetic regulation of cFLIP underlies resistance to TRAIL in pancreatic cancer**

Ji Hye Kim, Jinyoung Lee, Se Seul Im, Boyun Kim, Eun-Young Kim, Hyo-Jin Min, Jinbeom Heo, Eun-Ju Chang, Kyung-Chul Choi, Dong-Myung Shin, Jaekyoung Son

This PDF file includes:

Supplementary Figures 1 to 8 and figure legends

Supplementary Tables 1

**Supplementary Figures and Legends**

**Supplementary Figure 1**


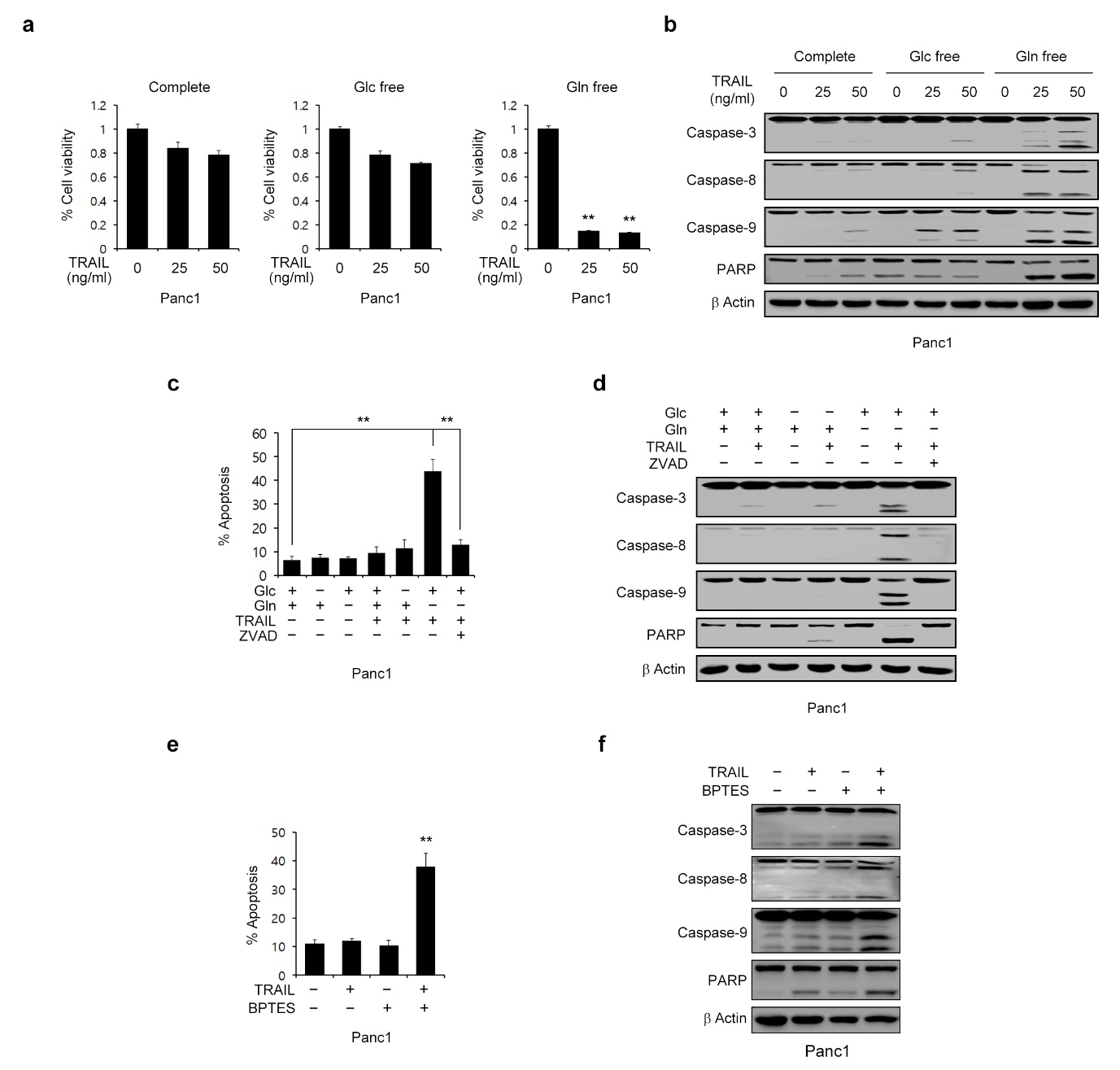


**Supplementary Fig. 1 Glutamine confers PDAC cells resistance to TRAIL.** **a and b** Panc1 cells were plated in complete medium. The next day, the medium was replaced with glucose- or glutamine-free medium and the cells were incubated for an additional 24 h followed by treatment with TRAIL at the indicated concentration for 4 h and cell viability assays (a); lysates were immunoblotted with the indicated antibodies (b). Error bars represent the s.d. of triplicate wells from a representative experiment. **c and d** Panc1 cells were plated in complete medium. The next day, the medium was replaced with glucose- or glutamine-free medium and the cells were incubated for an additional 24 h. Cells were treated with TRAIL at the indicated concentration for 4 h with or without zVAD-fmk (50 μM). Cell death was assessed by Annexin V/PI staining and flow cytometry (c) and lysates were immunoblotted with the indicated antibodies (d). Error bars represent the s.d. of triplicate wells from a representative experiment. **e and f** Panc1 cells were treated with BPTES (20 μM) for 24 h and then treated with TRAIL (50 ng/mL) for 4 h. Cell death was assessed by Annexin V/PI staining and flow cytometry (e) and lysates were immunoblotted with the indicated antibodies (f). Error bars represent the s.d. of triplicate wells from a representative experiment. ** *P*<0.01.

**Supplementary Figure 2**


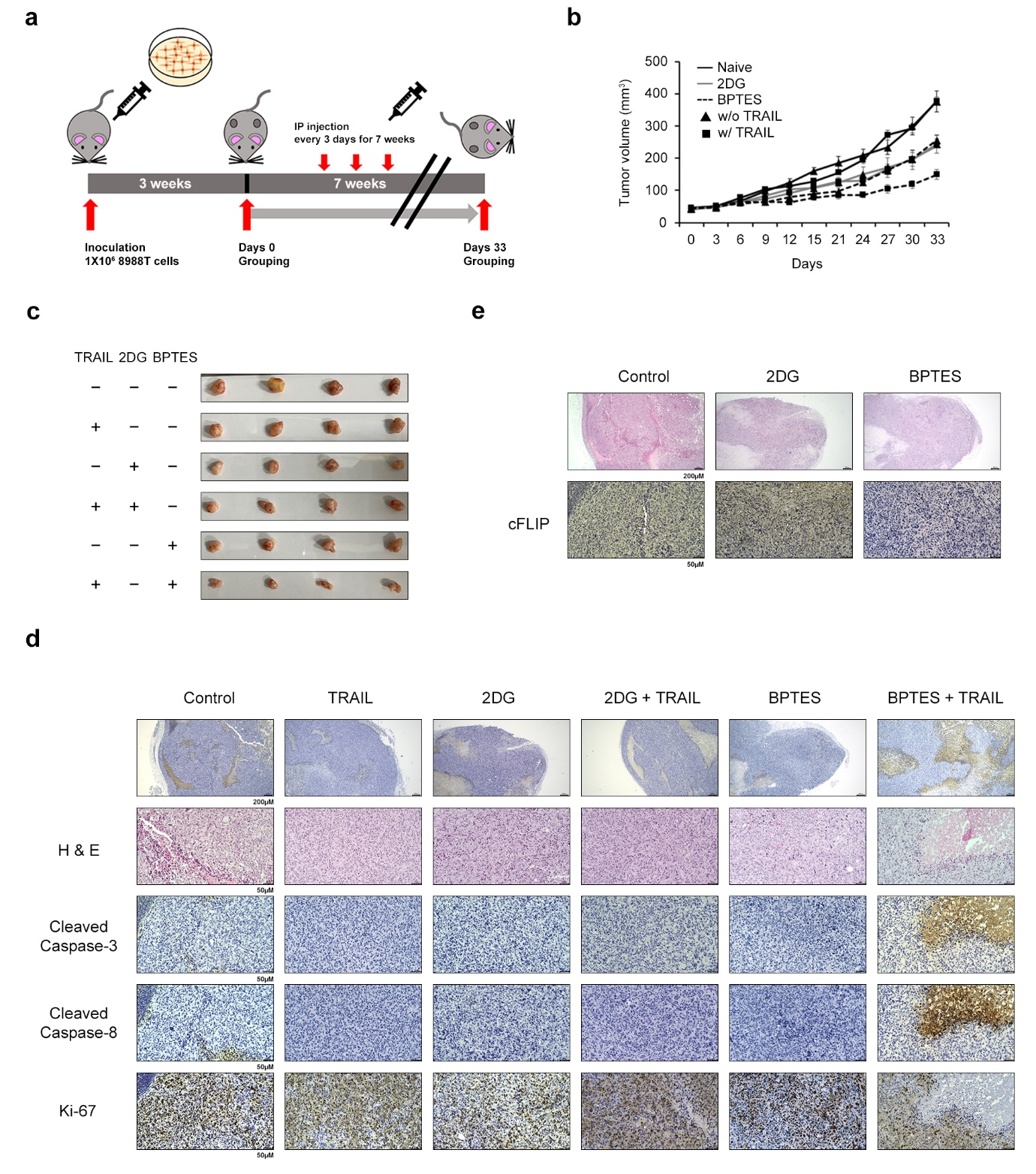


**Supplementary Fig. 2 The synergistic antitumor effect of the combination of TRAIL and BPTES**. **a** Schematic depiction of the animal experimental setup. Randomized 8988T cell-bearing mice were treated with 2DG (500 mg/kg/day) or BPTES (12.5 mg/kg/day) and with/without TRAIL (5 µg/kg/day) for 33 days. **b** Tumor volumes were calculated on the indicated days. Error bars represent s.e.m. **c** Representative images of the xenograft tumors obtained from the mice. **d** Representative sections from each xenograft tumor were stained with hematoxylin and eosin (H&E). Representative images of immunohistochemistry (IHC) for the indicated antibodies are shown for the paired samples. IHC was performed using tissues from the same passage number xenograft used for the H&E images (scale bars, 50 µm). **e** The expression of cFLIP was determined in tissue sections from xenograft tumors using IHC (scale bars, 50 µm)

**Supplementary Figure 3**

**
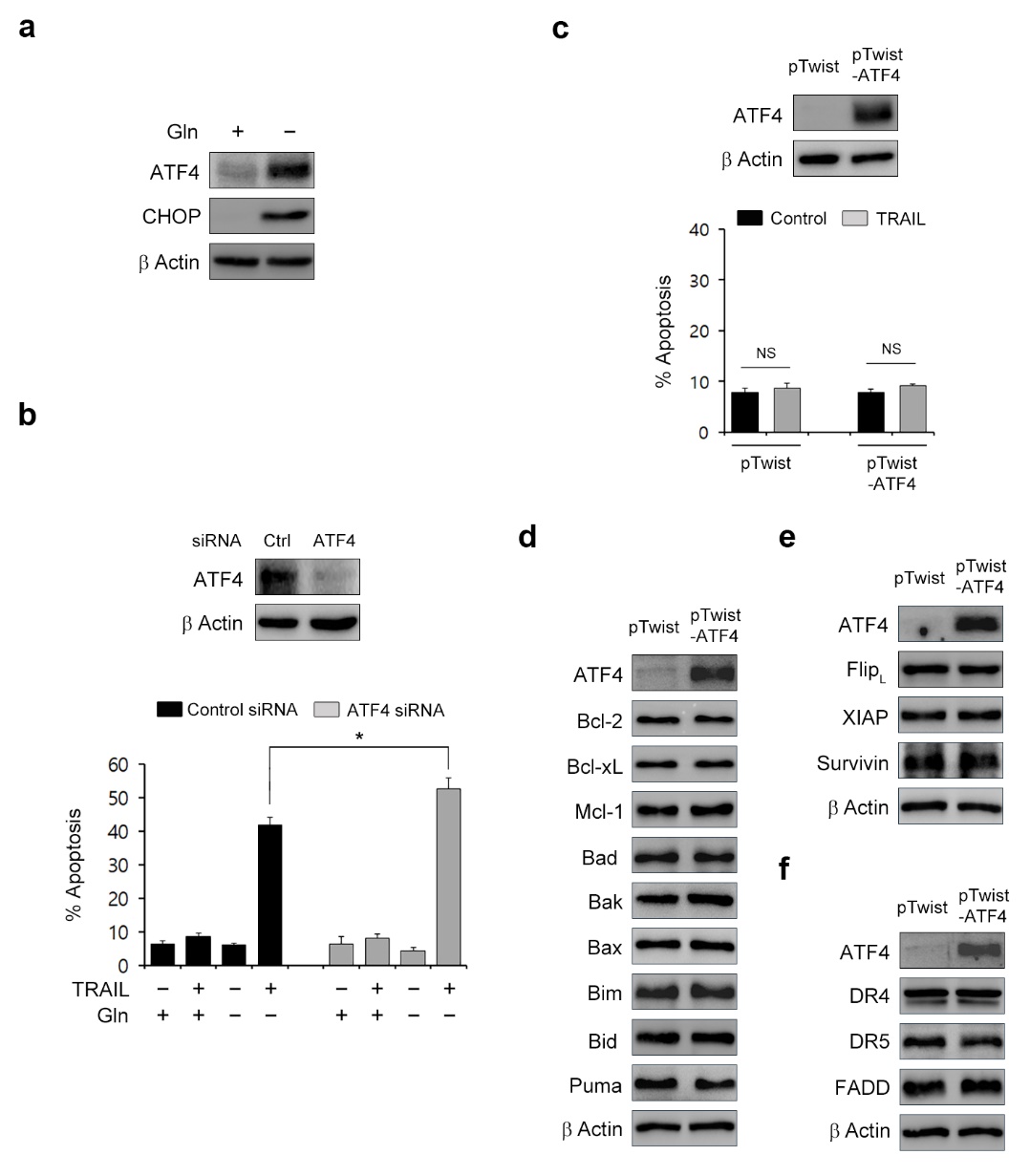
**

**Supplementary Fig. 3 ATF4 does not play a critical role in TRAIL-induced apoptosis**. **a** 8988T cells were plated in complete medium, which was replaced the following day with glutamine-free medium, incubated for an additional 24 h, and immunoblotted with the indicated antibodies. **b** 8988T cells expressing a control siRNA (siGFP) or siRNAs targeting ATF4 were treated with TRAIL (50 ng/mL) for 4 h, and cell death was assessed by Annexin V/PI staining and flow cytometry. **c** 8988T cells expressing pTwist (empty vector) or pTwist-ATF4 (ATF4) were treated with TRAIL (50 ng/mL) for 4 h, and cell death was assessed by Annexin V/PI staining and flow cytometry. **d, e and f** 8988T cells expressing pTwist (empty vector) or pTwist-ATF4 (ATF4) were immunoblotted with the indicated antibodies. Error bars represent the s.d. of triplicate wells from a representative experiment NS, not significant. * *P*<0.05.

**Supplementary Figure 4**


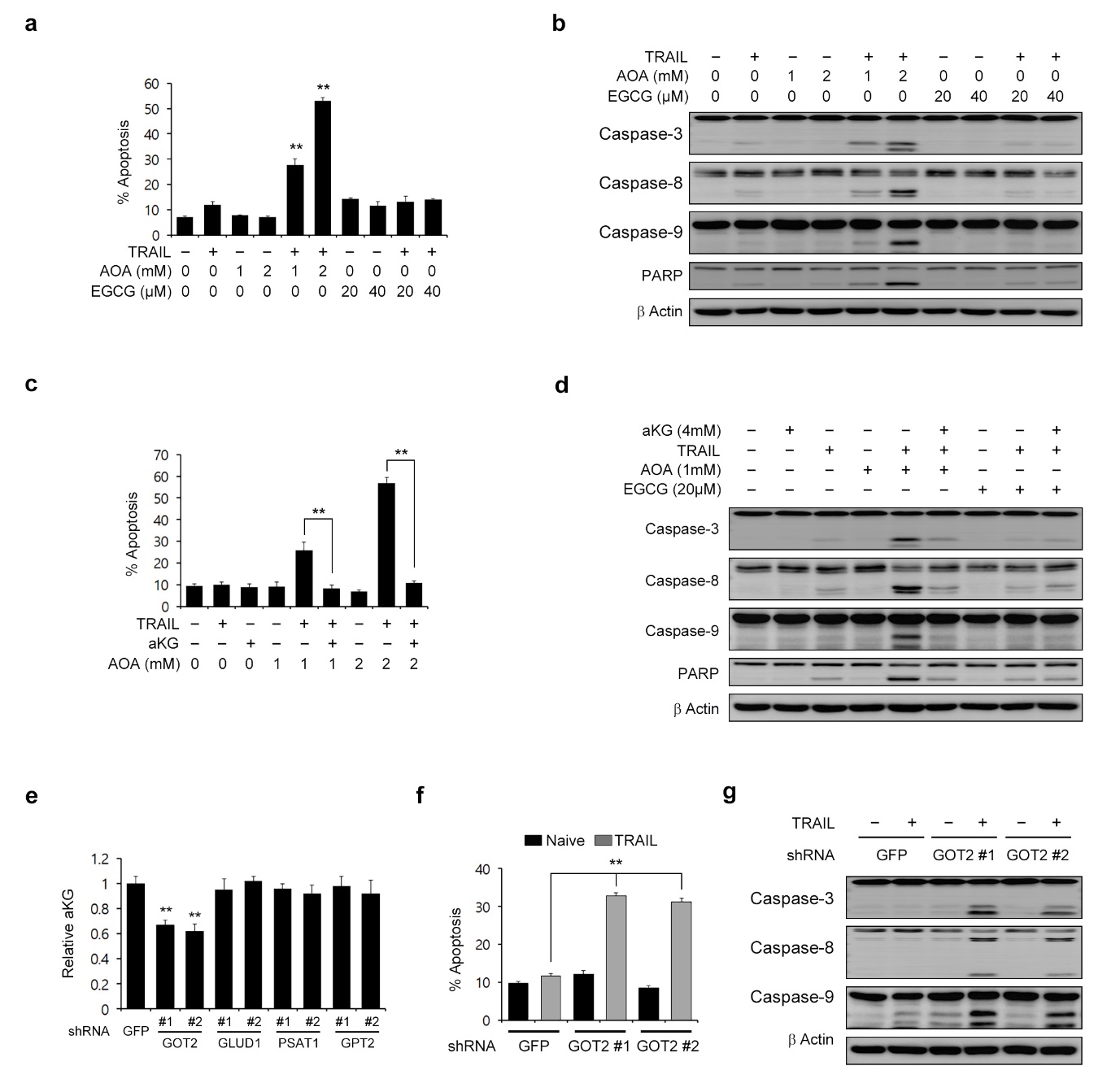


**Supplementary Fig. 4 GOT2-mediated aKG production is important for the regulation of cFLIP levels**. **a and b** Panc1 cells were treated with AOA or EGCG at the indicated doses for 24 h and treated with TRAIL (50 ng/mL) for 4 h. Cell death was assessed by Annexin V/PI staining and flow cytometry (a) and lysates were immunoblotted with the indicated antibodies (b). Error bars represent the s.d. of triplicate wells from a representative experiment. **c** Panc1 cells were treated with AOA at the indicated doses for 24 h in the presence or absence of aKG (4 mM) and then treated with TRAIL (50 ng/mL). Cell death was assessed by Annexin V/PI staining and flow cytometry. Error bars represent the s.d. of triplicate wells from a representative experiment. **d** Panc1 cells were treated with AOA or EGCG at the indicated doses for 24 h in the presence or absence of aKG (4 mM) and then treated with TRAIL (50 ng/mL) for 4 h and immunoblotted with the indicated antibodies. **e** AKG metabolite levels in Panc1 cells expressing control shRNA (GFP), GOT2 shRNAs, GLUD1 shRNAs, PSAT1 shRNAs, or GPT2 shRNAs. Error bars represent the s.d. of triplicate wells from a representative experiment. **f and g** Panc1 cells expressing control shRNA (GFP) or GOT2 shRNAs were treated with TRAIL (50 ng/mL) and cell death was assessed by Annexin V/PI staining and flow cytometry (f). Lysates were immunoblotted with the indicated antibodies (g). Error bars represent the s.d. of triplicate wells from a representative experiment. ** *P*<0.01.

**Supplementary Figure 5**

**
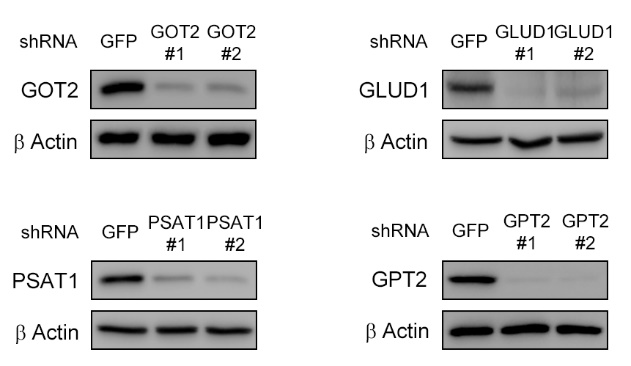
**

**Supplementary Fig. 5 Western blot confirms knockdown of Glu-dependent transaminases**. **a, b, c and d** Expression of GOT2, GLUD1, PSAT1 and GPT2 was determined by western blot analysis of 8988T cells expressing control shRNA (GFP), GOT2 shRNAs, GLUD1 shRNAs, PSAT1 shRNAs, or GPT2 shRNAs.

**Supplementary Figure 6**


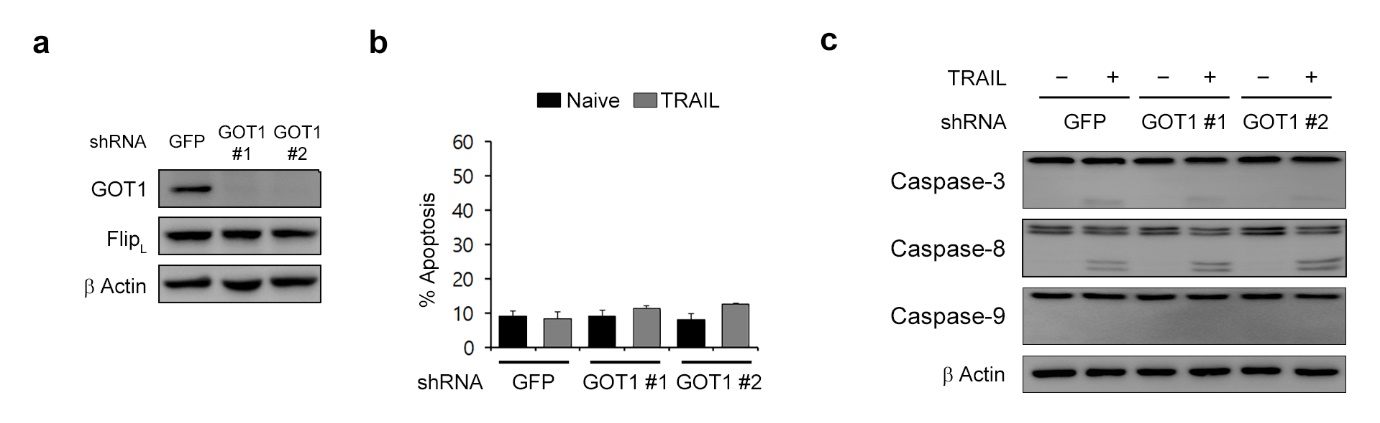


**Supplementary Fig. 6 GOT1 is not critical for the regulation of cFLIP levels**. **a** 8988T cells expressing control shRNA (GFP), or GOT1 shRNAs were immunoblotted with the indicated antibodies. **b** **and c** 8988T cells expressing control shRNA (GFP) or GOT1 shRNAs were treated with TRAIL (50 ng/mL) and cell death was assessed by Annexin V/PI staining and flow cytometry (b). Lysates were immunoblotted with the indicated antibodies (c). Error bars represent the s.d. of triplicate wells from a representative experiment.

**Supplementary Figure 7**


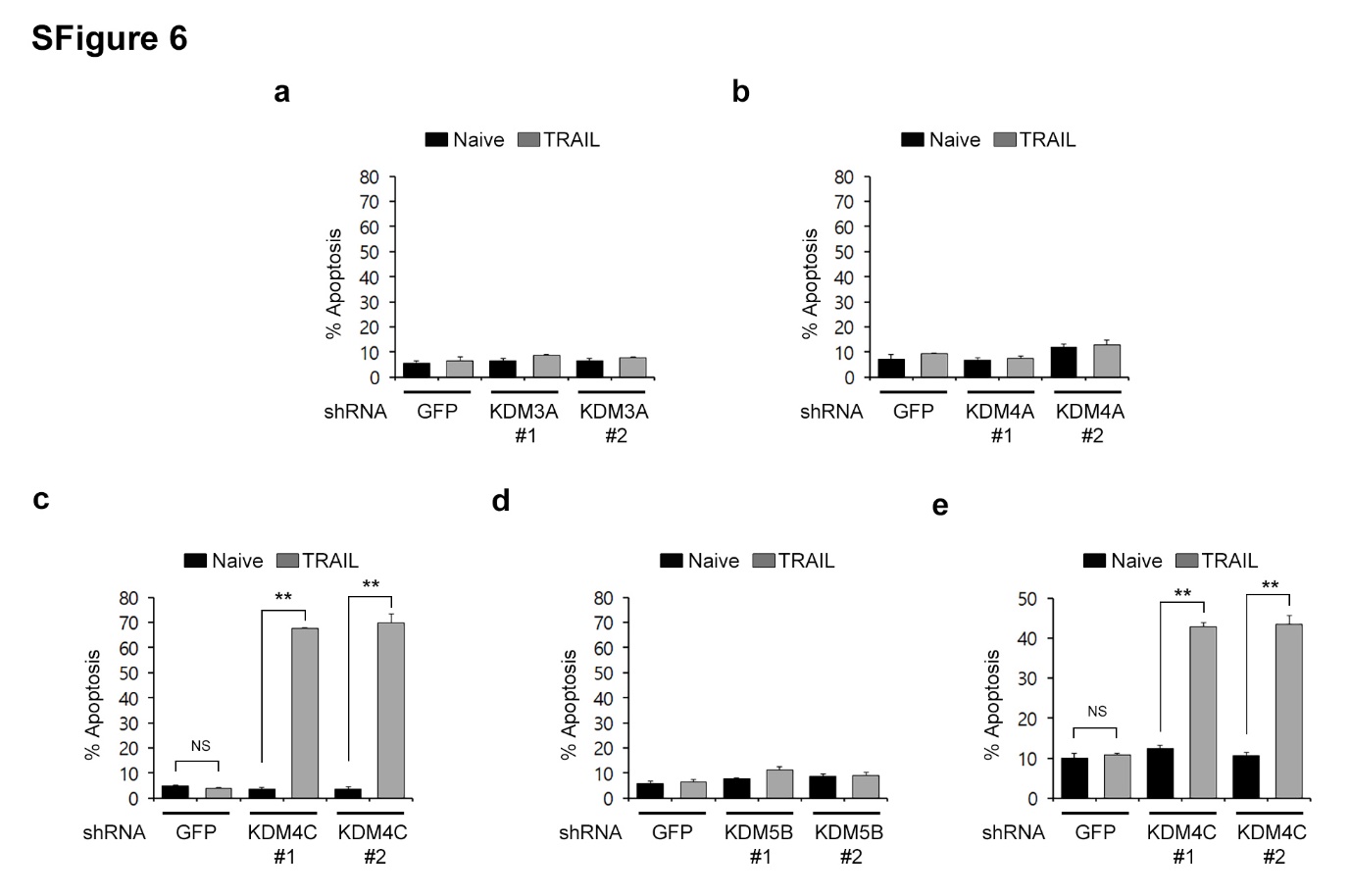


**Supplementary Fig. 7 Knockdown of KDM4c triggers apoptotic cell death following TRAIL treatment**. **a, b, c and d** 8988T cells expressing control shRNA (GFP), KDM3A shRNAs, KDM4A shRNAs, KDM4C shRNAs, or KDM5B shRNAs were treated with TRAIL (50 ng/mL) and cell death was assessed by Annexin V/PI staining and flow cytometry. **e** Panc1 cells expressing control shRNA (GFP) or KDM4C shRNAs were treated with TRAIL (50 ng/mL) and cell death was assessed by Annexin V/PI staining and flow cytometry. Error bars represent the s.d. of triplicate wells from a representative experiment. ** *P*<0.01.

**Supplementary Figure 8**


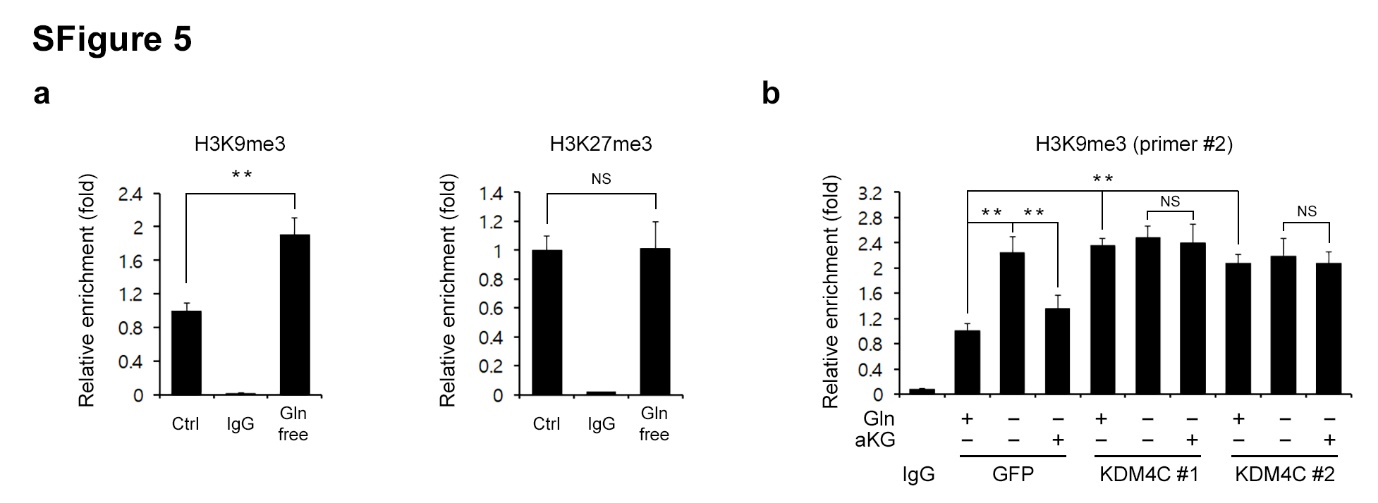


**Supplementary Fig. 8 Methylation of histone H3 lysine**. **a** 8988T cells were plated in complete medium that was replaced the following day with glutamine-free medium and cells were incubated for an additional 24 h. Chromatin was prepared for H3K9me3 or H3K27me3. **b** Chromatin was prepared for H3K9me3 in 8988T cells expressing control shRNA (GFP) or KDM4C shRNAs. ChIP analysis compared with isotype control (Ctrl). ChIP eluates were amplified by Q-PCR for the indicated regions of the cFLIP gene locus. Error bars represent the s.d. of triplicate wells from a representative experiment. ** *P*<0.01.

**Supplementary Tables**

**Supplementary Table 1. Oligonucleotides for shRNA**

| Oligonucleotides (shRNA) | TRC number |
| --- | --- |
| Human cFLIP #1 | TRCN0000007229 |
| Human cFLIP #2 | TRCN0000320670 |
| Human GOT1 #1 | TRCN0000034784 |
| Human GOT1 #2 | TRCN0000034785 |
| Human GOT2 #1 | TRCN0000034824 |
| Human GOT2 #2 | TRCN0000034825 |
| Human GLUD1 #1 | TRCN0000220878 |
| Human GLUD1 #2 | TRCN0000220880 |
| Human PSAT1 #1 | TRCN0000035264 |
| Human PSAT1 #2 | TRCN0000035265 |
| Human GPT2 #1 | TRCN0000035024 |
| Human GPT2 #2 | TRCN0000035025 |
| Human TET1 #1 | TRCN0000075024 |
| Human TET1 #2 | TRCN0000075025 |
| Human TET2 #1 | TRCN0000418976 |
| Human TET2 #2 | TRCN0000421134 |
| Human TET3 #1 | TRCN0000246257 |
| Human TET3 #2 | TRCN0000246260 |
| Human KDM3A #1 | TRCN0000329990 |
| Human KDM3A #2 | TRCN0000329992 |
| Human KDM4A #1 | TRCN0000013495 |
| Human KDM4A #2 | TRCN0000234910 |
| Human KDM4C #1 | TRCN0000235047 |
| Human KDM4C #2 | TRNC0000022054 |
| Human KDM5B #1 | TRCN0000358504 |
| Human KDM5B #2 | TRCN0000329951 |
